# Edge-Orientation Entropy Predicts Preference for Diverse Types of Man-Made Images

**DOI:** 10.3389/fnins.2018.00678

**Published:** 2018-09-28

**Authors:** Maria Grebenkina, Anselm Brachmann, Marco Bertamini, Ali Kaduhm, Christoph Redies

**Affiliations:** ^1^Experimental Aesthetics Group, Institute of Anatomy I, Jena University Hospital, School of Medicine, University of Jena, Jena, Germany; ^2^Department of Psychological Sciences, University of Liverpool, Liverpool, United Kingdom

**Keywords:** experimental aesthetics, aesthetic rating, visual preference, image properties, curved/angular stimuli, luminance edges

## Abstract

We recently found that luminance edges are more evenly distributed across orientations in large subsets of traditional artworks, i.e., artworks are characterized by a relatively high entropy of edge orientations, when compared to several categories of other (non-art) images. In the present study, we asked whether edge-orientation entropy is associated with aesthetic preference in a wide variety of other man-made visual patterns and scenes. In the first (exploratory) part of the study, participants rated the aesthetic appeal of simple shapes, artificial ornamental patterns, facades of buildings, scenes of interior architecture, and music album covers. Results indicated that edge-orientation entropy predicts aesthetic ratings for these stimuli. However, the magnitude of the effect depended on the type of images analyzed, on the range of entropy values encountered, and on the type of aesthetic rating (*pleasing, interesting*, or *harmonious*). For example, edge-orientation entropy predicted about half of the variance when participants rated facade photographs for *pleasing* and *interesting*, but only for 3.5% of the variance for *harmonious* ratings of music album covers. We also asked whether edge-orientation entropy relates to the well-established human preference for curved over angular shapes. Our analysis revealed that edge-orientation entropy was as good or an even better predictor for the aesthetic ratings than curvilinearity. Moreover, entropy could substitute for *shape*, at least in part, to predict the aesthetic ratings. In the second (experimental) part of this study, we generated complex line stimuli that systematically varied in their edge-orientation entropy and curved/angular shape. Here, edge-orientation entropy was a more powerful predictor for ratings of *pleasing* and *harmonious* than curvilinearity, and as good a predictor for *interesting*. Again, the two image properties shared a large portion of variance between them. In summary, our results indicate that edge-orientation entropy predicts aesthetic ratings in diverse man-made visual stimuli. Moreover, the preference for high edge-orientation entropy shares a large portion of predicted variance with the preference for curved over angular stimuli.

## Introduction

Since the inception of experimental aesthetics by Gustav Theodor Fechner (1801–1887), one of the central goals in this field of research has been to identify objective physical properties of images that humans perceive as visually pleasing (Fechner, 1876). It is assumed that at least some of these properties are universal across different cultures and relate to basic mechanisms of information processing in the human visual system (Bell, 1914; Berlyne, 1974; Spehar et al., 2003; Redies, 2007, 2015).

With the advent of powerful technologies in image processing and analysis, increasingly complex image properties and their association with aesthetic perception have been investigated (for reviews, see Graham and Redies, 2010; Brachmann and Redies, 2017). Examples are visual complexity (Birkhoff, 1933; Berlyne, 1974; Jacobsen and Höfel, 2002; Forsythe et al., 2011; Güclütürk et al., 2016), a scale-invariant Fourier spectrum (Graham and Field, 2007; Redies et al., 2007), symmetry (Bertamini and Makin, 2014; Wright et al., 2017), and fractality and self-similarity (Taylor, 2002; Spehar et al., 2003; Taylor et al., 2011; Redies et al., 2012; Braun et al., 2013). The present work focuses on two additional stimulus properties that have been associated with visual preference, curvi-/rectilinearity (the preference of curved over angular shapes; Bar and Neta, 2006), and edge orientation entropy (Redies et al., 2017).

The preference of human observers for curved shapes over angular shapes is particularly striking and robust (for recent reviews, see Bertamini et al., 2016; Gómez-Puerto et al., 2016). Bar and Neta (2006) proposed that angularity reflects potential danger and is thus perceived as threatening (and hence less attractive). Palumbo et al. (2015) confirmed that smoothly curved shapes are rated as more pleasant than angular ones. They studied approaching and avoiding reactions to curved and angular polygons and presented evidence that the preference for curved over angular forms does not result from a perceived threat of angular forms (Bar and Neta, 2006), but rather from a preference for the curved ones. Other studies have since confirmed this preference (Bertamini et al., 2016; Velasco et al., 2016). For example, Gómez-Puerto et al. (2017) investigated responses to curved/angular patterns by human observers in three different regions (Mexico, Ghana, and Mallorca) and concluded that the preference for curvature is common across these cultures. The preference for curved objects can even be observed in primates like chimpanzees and gorillas, but is smaller than in humans (Munar et al., 2015). The authors proposed that the human preference for curved objects is an evolutionarily ancient trait, but became modulated by higher cognitive processes and other visual preferences during human evolution. Blazhenkova and Kumar (2018) asked participants to match curved/angular shapes with five different sensory modalities and higher-level attributes, such as gender, emotion and name, and obtained non-arbitrary mappings as a result (see also the earlier work by Poffenberger and Barrows, 1924; Hevner, 1935). For example, curved shapes were associated to sweet taste and smooth texture as well as female gender and relieved emotion, while angular shapes were linked to spicy smell, rough texture, male gender and excited emotion.

Although the preference for curved objects and forms over angular ones is well established and widely recognized, fundamental questions remain, as pointed out by Gómez-Puerto et al. (2016). In particular, the terminology is variable and most terms used in the field (curved, straight, sharp, angular, waving etc.) lack a physical definition that would allow an objective quantification. Also, it is unclear how the curved/angular account of visual preference relates to other image properties that have been associated with visually preferred stimuli.

To address these shortcomings in the present work, we draw a connection between curvilinearity and Shannon edge-orientation entropy. We have recently shown that artworks possess a high entropy of edge orientations compared to many categories of non-art images, i.e., luminance gradients and edges are more evenly distributed across orientations in artworks (Redies et al., 2017). This result holds for large subsets of traditional artworks from different cultural provenance.

Regularities in edge orientations across images of natural objects and scenes have been studied before, in particular in photographs of natural scenes (Geisler et al., 2001; Sigman et al., 2001). Recently, Redies et al. (2017) showed that there are many types of natural growth patterns, such as photographs of lichen and plants, which possess a relatively high degree of edge-orientation entropy. In many of these growth patterns, local oriented edges have emerged more or less randomly and independently so that all edge orientations are about equally likely to occur (high 1st-order entropy) and there are few correlations of edge orientations across the images (high 2nd-order entropy). In other natural stimuli, edge-orientation entropy can be relatively low, for example in some large-vista natural scenes where the horizon and vertical growth patterns (e.g., of trees) can induce regularities so that cardinal orientations are more dominant and correlate across the images. Lower edge-orientation entropy was also observed in photographs of simple objects and faces (Redies et al., 2017).

It remains unclear, however, whether man-made visual stimuli other than artworks also exhibit high edge-orientation entropy and whether this image property can be related to aesthetic perception more generally (Redies et al., 2017). To answer this question, the present study explored a wide spectrum of man-made visual stimuli and patterns. We hypothesize that edge orientation entropy is a predictor for aesthetic ratings in at least some of these stimuli as well.

**FIGURE 1:**
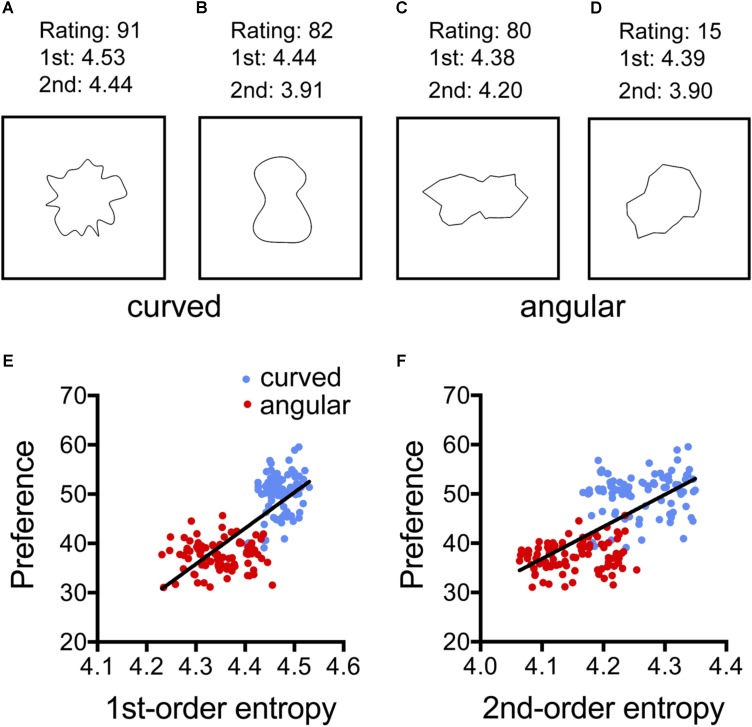
Results from the analysis of the study by Bertamini et al. (2016; Experiment 1). **(A–D)** Examples of the images used. **(E,F)** Dot plots of the relation between preference and 1st-order entropy **(E)** and 2nd-order entropy of edge orientations **(F)**. Each dot represents the results for one type of pattern, averaged across participants. The straight lines in **(E,F)** indicate the results from simple regression analyses (**E**, *R*^2^ = 0.563; *F*[1,178] = 229.1, *p* < 0.0001; and **F**, *R*^2^ = 0.465; *F*[1,178] = 154,7, *p* < 0.0001). 1st, 1st-order entropy; 2nd, 2nd-order entropy.

Moreover, we investigated the relation between edge-orientation entropy and curvilinearity. Such a relation seems obvious if one considers simple shapes, such as the stimuli used by Bertamini et al. (2016) in their study of preference for curved over angular shapes (**Figures 1A–D**). If the shapes are angular, straight lines predominate and the orientations of luminance edges tend to be less evenly distributed than in similar shapes that are composed of curved lines. We therefore expected and indeed found in Experiment 1 that edge orientations are more evenly distributed across orientations for the curved patterns than for the angular versions in these stimuli (**Figures 1E,F**). Based on this scenario, we hypothesized that human observers prefer a high degree of edge-orientation entropy in more complex visual stimuli, including photographs of man-made objects and scenes, as well. Moreover, we speculated that edge-orientation entropy and curvilinearity share predicted variance of preference for visual stimuli.

To test the above hypotheses, we asked participants to give ratings of their aesthetic preference for various sets of man-made images that differ in their curvilinearity or edge-orientation entropy. We thus investigated a wide variety of visual stimuli, ranging from simple line patterns to more complex abstract patterns and photographs of common man-made patterns and scenes (for an overview, see **Table 1**). For the present synopsis, we focused on group-level differences and, as a consequence, did not address the issue of possible inter-individual differences in aesthetic ratings (Berlyne, 1971; Jacobsen and Höfel, 2002; Palmer and Griscom, 2012; Mallon et al., 2014; Güclütürk et al., 2016; Spehar et al., 2016).

**Table 1:** Overview of experiments.

Experiment.	Type of study	Type of Stimulus	Examples shown in	Number of stimuli	Number of participants	Rating term(s)	Curvilinearity variable	Results listed in	Original rating data from
1	Exploratory	Simple artificial shapes	**Figure 1**	3600	20	*Liking*	*shape* (curved/angular)	**Table 2** and **Figure 1**	Bertamini et al., 2016
2	Exploratory	Complex artificial patterns (Taprats ornaments)	**Figure 2**	100	31	*Interesting/pleasing/harmonious*	None	**Table 3**, **Figure 2**, and **Supplementary Table S1**	Present study
3	Exploratory	Photographs of complex man-made objects (building facades)	**Figure 3**	50	27	*Interesting/pleasing/harmonious*	None	**Table 4**, **Figure 3**, and **Supplementary Table S2**	Present study
4	Exploratory	Photographs of complex man-made scenes (interior architecture)	**Figure 4**	200	18	*Pleasantness/beauty*	*Contour* (curvilinear/rectilinear)	**Table 5**, **Figure 4**, and **Supplementary Table S3**	Vartanian et al., 2013
5	Exploratory	Complex man-made design (music album covers)	**Figure 5**	150	27 (same as in Experiment 3)	*Interesting/pleasing/harmonious*	None	**Table 6**, **Figure 5**, and **Supplementary Table S4**	Present study
6	Experimental	Complex artificial line patterns	**Figure 7**	100	31 (same as in Experiment 2)	*Interesting/pleasing/harmonious*	*Shape* (curved/angular)	**Table 7**, **Figures 6**, **8**, and **Supplementary Table S5**	Present study

In our study of images of man-made patterns and scenes, we included stimuli that were produced to be preferred by human observers, such as interior scenes or music album covers. Moreover, we studied images that are abstract as well as images that display recognizable content. We expected that formal image properties would have a smaller effect on aesthetic ratings that are driven by image content.

Specifically, the following five sets of images were analyzed:

(1) Simple irregular geometric shapes with a black contour on a white background (Experiment 1). The shapes were taken from the study by Bertamini et al. (2016) and consisted of either curved lines (for examples, see **Figures 1A,B**) or straight lines (**Figures 1C,D**). In a rating study, the authors revealed that participants liked the curved patterns more than the angular ones (see above). In the present study, we re-analyzed the data from Bertamini et al. (2016) and related them to image properties.
(2) As an example of complex artificial patterns, we generated ornamental stimuli that are reminiscent of Islamic decorative art, by using the program *Taprats* (Experiment 2; Kaplan, 2000). Besides varying their curvilinearity, the patterns were designed to display different degrees of complexity and self-similarity. These two image properties have been previously associated with the preference for visual stimuli, including artworks (Birkhoff, 1933; Berlyne, 1974; Jacobsen and Höfel, 2002; Forsythe et al., 2011; Taylor et al., 2011; Braun et al., 2013). In the present study, we therefore asked whether there is an overlap between the contribution of these two independent variables and edge orientation entropy.

In addition, we analyzed the following datasets and asked whether edge-orientation entropy has an effect on the preference of man-made patterns and scenes that surround us in every-day life:

(3) Photographs of building facades, which range from rather simple window patterns of mostly horizontal and vertical edge orientations, to facades that were highly ornamental (Experiment 3). Facades represent a type of images that are affectively neutral, or at least with a low arousal potential, but can be more or less pleasing to human observers.
(4) Interior architectural scenes that differ in their curvilinearity as well as in other architectural impressions (openness and ceiling height; Experiment 4). These stimuli were introduced and rated for their beauty and pleasantness in a previous study (Vartanian et al., 2013), which provided behavioral and neurophysiological evidence that the aesthetic preference for curvilinearity can be extended to architecture. We related these previous rating data to the image properties analyzed in the present study.
(5) Album covers from three different music genres (classic, pop, and metal) served as examples of man-made images that vary in artistic demand and graphic design (Experiment 5). It has been shown that the presence of visual arts can enhance the evaluation of consumer perceptions and evaluations of products with which it is associated (Hagtvedt and Patrick, 2008). Moreover, album cover artwork can encode valuable information that helps place an artist into a musical context (Libeks and Turnbull, 2011). Here, we asked whether curvilinearity can possibly influence visual preference of album covers and might thus be a factor that can potentially affect consumer perception of music albums.

After the analysis of these datasets, we carried out a more direct test of the predictive power of edge-orientation entropy (Experiment 6). We created a set of artificial stimuli that are composed of identical sets of either curved or angular line elements and used them in an aesthetic rating experiment, in which the lines were arranged so that they differed in curvilinearity or edge-orientation entropy. This experimental design allowed us to study the contribution of the two factors on the aesthetic ratings.

In all experiments (except for the previously rated stimuli of Experiments 1 and 4; **Table 1**), we asked participants to evaluate the images according to three aesthetic rating terms (*pleasing*, *interesting*, and *harmonious*). We chose these terms based on a study by Cupchik and Gebotys (1990), which revealed differences between the categories *interesting* and *pleasing*. The authors carried out multidimensional scaling and found that *interesting* relates to the dimensions of complexity and familiarity of images, whereas *pleasing* reflects the dimensions of emotional arousal and aesthetic effectance. Another model, proposed by Marković (2012), identified two similar factors: aesthetic experience and hedonic tone. The former was related to ratings of “interesting,” “complex,” “imaginative,” whereas hedonic tone was more related to ratings of “pleasant,” “cheerful,” “warm.” In addition, we used *harmonious* as a term that relates more clearly to the hedonic value derived from image composition (Redies et al., 2015).

In multiple regression analyses, we related the ratings to the 1st-order and 2nd-order measures of the statistical distribution of edge orientations (Redies et al., 2017). In Experiments 1, 4, and 6, we included the angular/curved classification of the stimuli as additional predictors for their preference, as well as two other measures, self-similarity and edge density, which have been associated with traditional artworks previously (Braun et al., 2013). Self-similarity is a measure of how similar the histogram of oriented gradients are in parts of an image, compared to the histogram of the entire image (Dalal and Triggs, 2005; Amirshahi et al., 2012). Edge density is calculated on the basis of Gabor-filter responses (Redies et al., 2017) and served as a measure that is related to subjective ratings of complexity in images (Berlyne, 1971; Taylor et al., 2011; Braun et al., 2013). We included these properties as predictors of aesthetic preference in multiple regression analyses that evaluate their contribution to predicting the aesthetic ratings, relative to edge orientation entropy and curvilinearity.

Our study thus allows a comprehensive analysis of the role of curvilinearity and edge-orientation entropy in diverse types of man-made stimuli along different dimensions of visual preference.

## General Methods

### Image Analysis

The analysis of objective image properties followed previously published procedures. In brief, the following properties were calculated:

#### Entropy of Edge Orientations

To study the spatial distribution of oriented edges in each image, we calculated the 1st-order and the 2nd-order Shannon entropy of orientation histograms (Geisler, 2008), as modified by Redies et al. (2017). Edge-orientation entropy should not be confused with the Shannon entropy of gray level values. Entropy of gray level values refers to the probability of encountering particular gray level values across an image (e.g., Kersten, 1987) or in local patches of an image (e.g., Chandler and Field, 2007). In contrast, edge-orientation entropy is based on edge-filtered images and refers to the probability of encountering particular orientations in an image (1st-order entropy) or to the statistics of pairwise comparisons of edge orientations across an image (2nd-order entropy; Geisler et al., 2001; Redies et al., 2017).

Briefly, we first downscaled the original input images to a size of 120,000 pixels. All color images were converted to grayscale images by using the ITU-R-601-2 luma transform, which weights the color channels according to their perceived luminosity. To extract edges, a set of 24 oriented Gabor filters that represented one full circle was applied (for details of the calculations, see Redies et al., 2017). Due to computing power restrictions, only the 10,000 highest edge responses were included in the analysis.

##### First-order edge-orientation entropy

First-order edge-orientation entropy was defined as the Shannon entropy for the summary orientation histogram that represented the strength of all edge orientations for the entire image. It measured how uniformly the edge orientations were distributed across the full spectrum of orientations in each image (Redies et al., 2017). Entropy was higher for more uniform orientation histograms, i.e., if all orientations were present at about equal strength in the image, and lower for unevenly distributed histograms. The maximum value for 24 bins of orientations (Gabor filters) was about 4.585.

##### Second-order edge-orientation entropy

Second-order edge-orientation entropy was calculated to measure how independent edge orientations were across an image. To obtain this measure, the orientation of each edge element was related to the orientation of all other edge elements in the same image by pairwise comparison (Geisler et al., 2001; Redies et al., 2017). First, for each edge pair, the orientation of the first (reference) edge was normalized to be horizontal. Then, for each distance *d* and radial direction *α* between all edge pairs, we obtained a 1*d* histogram, as we summed up the relative orientations *θ* between all edge pairs (*θ* histograms). For each bin defined by *d* (500 bins) and *α* (48 bins), histograms of the angles *θ* (24 bins across the full circle of orientations) were normalized. The *θ* histograms indicate the weighted probability, *P(d,α,θ)*, of observing an edge element at distance *d* in direction *α* and with an orientation difference *θ* for any given (reference) edge element. Filter responses near the image border (15 pixels absolute) were not included in the analysis. As a measure for the uniformity of the *θ* histograms, we calculated the Shannon entropy H, as follows:

(1) $$H(X)=-\sum_{\text{i}=1}^{\text{n}}p(x_{\text{i}})\cdot \log_{2}p(x_{\text{i}})$$

where *X* is the *θ* histogram at distance *d* and angle *α*. For the 24 bins in the *θ* histogram, the theoretical entropy maximum is about 4.585 (same as for 1st-order entropy). Entropy values close to this maximum indicate a high degree of uniformity in a *θ* histogram, i.e., all orientations encountered in a bin at distance *d* and angle *α* relative to the orientation of the reference edge are about equally likely to occur, meaning that edge orientations are independent of each other. Entropy is lower for less uniform histograms, in which particular orientations are more prevalent compared to others. If 2nd-order entropy is low, orientation of one edge predicts orientation of other edges in the image with some non-random probability. To simplify the quantification of the results, we plotted entropy as a function of distance *d* by averaging 2nd-order entropy across directions *α*. Finally, we averaged the values in these 1*d* plots for the distance range from 20 to 240 pixels (except for Experiment 1). Distances below 20 pixels was omitted to exclude regions of local collinearity (Redies et al., 2017).

Images of traditional artworks of different cultural provenance have relatively high 1st-order entropy (Redies et al., 2017), indicating that all orientations are about equally prominent in artworks on average (see also Koch et al., 2010). Moreover, 2nd-order entropy is also high in traditional artworks. This result implies that the orientations of distant edge pairs are independent of each other (Redies et al., 2017). Note that 1st-order entropy and 2nd-order entropy are not independent of each other; in general, 2nd-order entropy can be high only if 1st-order entropy is high.

#### Edge Density

As a measure that relates to perceived complexity of an image, we summed up the responses of all Gabor filters across the entire image, i.e., we calculated its edge density. Note that this measure reflects not only the density of edges in an image but also their contrast (i.e., edge strength). Humans prefer visual patterns of intermediate visual complexity in general (Berlyne, 1974; Taylor et al., 2011), although there are large inter-individual differences in complexity preference (Bies et al., 2016; Güclütürk et al., 2016).

#### Self-Similarity of Gradient Orientations (PHOG Method)

For each image, we calculated self-similarity and anisotropy of oriented gradients with a method that was derived from the PHOG descriptor (Bosch et al., 2007), as described before (Amirshahi et al., 2012). Color images were transformed into the Lab color space and image size was uniformly reduced to 100,000 pixels by bicubic interpolation and isotropic scaling for all images in all categories (Braun et al., 2013). To obtain the PHOG descriptor, we then generated histograms of oriented gradients (HOG features; Dalal and Triggs, 2005) for each image at consecutive levels of an image pyramid (Bosch et al., 2007). Histograms were obtained for 16 equally sized bins covering the full circle (360 degrees; Braun et al., 2013). Initially, the HOG features were calculated first at the ground-level (level 0) for the entire image. Then, the image was divided into four rectangles of equal size (level 1) and HOG features were calculated for each of the four sections at this level. Each section at level 1 was then again divided into four rectangles of equal size to generate the next level of the pyramid, and so on. Level 2 thus comprised 16 sections and level 3 contained 64 sections. The HOG features were calculated for each section at a given level.

To obtain a measure of self-similarity, the histograms at different levels of the pyramid were compared with the ground-level histogram (Amirshahi et al., 2012). We calculated self-similarity as the mean value for levels 1–3 of the pyramid in the present study. At higher levels, values for self-similarity become unstable, due the exceedingly small size of the image sections that is used in the analysis (Amirshahi et al., 2012). A value of 0 indicates minimal self-similarity and a value close to 1 nearly complete self-similarity. For a detailed description of the procedure, see the Appendix in Braun et al. (2013).

### Rating Procedure

The stimuli and ratings analyzed in Experiments 1 and 4 were obtained from previous studies (Vartanian et al., 2013; Bertamini et al., 2016). For Experiments 2, 3, 5, and 6, the following general procedure was used to obtain aesthetic ratings on the set of stimuli used in each experiment.

#### Participants

Participants (for groups and numbers of participants, see **Table 1**) were recruited by advertisements in places frequented by students of various disciplines; they were mostly university students or graduates (for population details, see individual experiments). None of the participants were art professionals or had received formal training in the arts. All participants reported normal or corrected-to-normal vision. The study was approved by the ethics committee of Jena University Hospital (approval number 4808-05/16) and was carried out in accordance with the ethical guidelines of the Declaration of Helsinki. Prior to the experiment, all participants gave their written consent.

#### General Procedure

Before the experiments, participants were requested to fill out a questionnaire that included biographic questions (sex, age, handedness, and correction for abnormal vision; see individual experiments) as well as questions regarding the training and interest in the visual arts and music. Each experiment was carried out separately. Within each experiment, the subjective categories (*interesting, pleasing*, and *harmonious*) were evaluated separately as a block. For every participant, we randomized the order of the subjective categories as well as the order of the images within each block.

The stimuli were displayed on a black screen (EIZO ColorEdge CG241W) at a viewing distance of 70 cm that was assured by the use of a chin rest. Stimuli were presented at a size of 135 mm × 135 mm (10.92° × 10.92° of visual angle). For each stimulus run, a fixation cross was presented first (500 ms duration), followed by the stimulus image and a rating scale at the bottom of the screen. Participants were asked to rate each image on a continuous-looking, free scale (100 steps) that ranged from *not interesting* to *interesting* (and respective terms for *pleasing* and *harmonious*). The scale was displayed on the screen below the stimulus and participants entered their rating by clicking on the position chosen. For rating analysis, we converted the 100 steps of the rating scale to a scale ranging from 0 to 1. There was no time limitation for the participants to evaluate each image. After responding, the next presentation cycle began. Before starting the next evaluation block, participants were free to take a break for as long as they wished.

The stimuli used in Experiments 2, 3, and 6, which are not subject to copyright restrictions by third parties, as well as the raw data for all experiments can be retrieved from the Open Science Framework (accession code: osf.io/cxyj4).

### Statistical Methods

By multiple linear regression, we analyzed the dependence of the different ratings on the four calculated image properties (1st-order entropy, 2nd-order entropy, edge density, and self-similarity) for each experiment (Model 1). In Experiments 1 and 6, *shape* (curved or angular) served as an additional independent variable in Model 1, in Experiment 4 architectural features (curved or angular *contour*, *openness* of space and *ceiling height*), and in Experiment 5 music genre. We also considered reduced models that included the two measures of entropy as the only independent variables (Model 2). Moreover, for Experiments 1 and 6, we analyzed models that included the entropies and *shape* (Model 3) as well as *shape* alone (Model 4). This was done to assess the extent to which these variables of interest predicted the ratings, compared to the full models. Differences between models were assessed by an ANOVA (*R*^2^ difference test). Because the present study focuses on group-level differences, all ratings were averaged across participants before entering them in the analysis.

The analysis was carried out with the *lm* package in R (R Development Core Team, 2017). This program also calculated which of the variables had a significant effect on the rating when the other variables in a given model were controlled for (highlighted in bold letters in the tables). Moreover, using the same package, *R*^2^ values were calculated for each model to estimate how much of the variability in the outcome is accounted for by the predictors of each model. The *R*^2^ values were adjusted to account for the number of predictors in each model (*R*^2^_adj_). In addition, the package calculated the Akaike Information Criterion (*AIC*) which considers both the model fit and the number of parameters used; it allows to compare the relative quality of the fit for different models that are applied to the same set of data (Akaike, 1974). The smaller the *AIC* values, the better the model.

As an index for the effect of the variables on the outcome in the models, standardized regression coefficients β_i_ were calculated with the *lm.beta* package of the R project. This index estimates the number of standard deviations by which the outcome will change as a result of a change of one standard deviation in the predictor, provided that the effects of all other predictors are held constant (Field et al., 2012).

Additionally, the dependencies of the ratings on selected variables were visualized in scatter plots and are shown in the figures, together with the lines from simple linear regressions; statistics for these regressions are provided in the figure legends.

## Experiment 1: Patterns By Bertamini et al. (2016)

Bertamini et al. (2016) created a series of simple geometric patterns that consisted of either curved lines (for examples, see **Figures 1A,B**) or straight lines (**Figures 1C,D**). Their rating study revealed that participants preferred the curved patterns over the angular ones. Here, we asked whether the two types of patterns also differed in their edge-orientation entropy. This is likely because the angular patterns contain straight lines of specific orientations. As a consequence, the strength of the edges would tend to be less uniform across orientations for the angular than for the curved lines. We therefore anticipated that the participants of the rating study preferred the (curved) stimuli with the higher entropy.

### Methods

The 3,600 stimuli of Experiment 1 by Bertamini et al. (2016) were analyzed. The stimuli consisted of irregular shapes with a black contour on a white background. The shapes were based on Cassini ovals and were systematically altered along three parameters (shape, vertex, and articulation), resulting in 180 combinations of different stimulus parameters (90 with angular lines and 90 with curved lines; for *examples*, see **Figures 1A–D**). Image size was 460 pixels × 460 pixels. Bertamini et al. (2016) asked twenty participants to rate the 180 different combinations for preference on a scale ranging from *dislike* (0) to *like* (100) with a mouse click on a continuously looking scale presented on the screen below the stimulus. In the present study, the mean preference ratings from the study by Bertamini et al. (2016) for each combination of parameters were averaged over participants and were then entered in a multiple regression analysis in which *shape* (curved and angular) served as a binary variable to indicate curvilinearity (**Table 1**). Because the patterns typically covered only an area of about 250 pixels × 250 pixels in the images, the calculation of 2nd-order entropy was restricted to 20–160 pixels distance between edge pairs.

### Results

**FIGURE 2:**
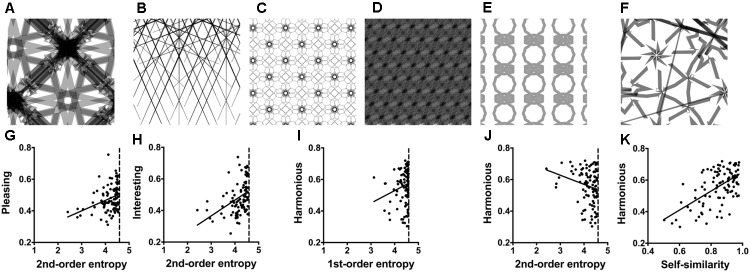
**(A–F)** Examples of the *Taprats* stimuli (Experiment 2). The ratings and image properties for the images are listed in **Supplementary Table S1**. **(G–K)** Dot plots of the relation between *pleasing* versus 2nd-order entropy of edge orientations **(G)**; *interesting* versus 2nd-order entropy **(H)**; *harmonious* versus 1st-order entropy **(I)**; *harmonious* versus 2nd-order entropy **(J)**; and *harmonious* versus self-similarity **(K)**. Each dot represents the results for one image, averaged across participants. The straight lines in **(G–K)** indicate the results from simple regression analyses (**G**, *R*^2^ = 0.113; *F*[1,98] = 12.51, *p* = 0.0006; **H**, *R*^2^ = 0.174; *F*[1,98] = 20.66, *p* < 0.0001; **I**, *R*^2^ = 0.037; *F*[1,98] = 3.73, *p* = 0.056; **J**, *R*^2^ = 0.048; *F*[1,98] = 4.91, *p* = 0.029; and **K**, *R*^2^ = 0.342; *F*[1,98] = 50.97, *p* < 0.0001).

**Figure 1** and **Table 2** summarize the results for the effect of the shape and the calculated image properties (entropies) on the mean preference ratings. **Table 2** lists the results for the four models that were used in the regression analysis. In the model with all independent variables (Model 1; see “Statistical Methods” section) and in Model 3, which includes *shape*, 1st-order entropy and 2nd-order entropy as independent variables, *shape* was the strongest predictor. In Model 2, which excluded *shape*, both edge-orientation entropies predicted preference, with 1st-order entropy being the strongest predictor. The explained variance (*R*^2^_adj_) was larger for Model 1 (75%) than for Model 2 (58%), and larger for Model 3 (75%) than for Model 2, but it did not differ significantly between any of the other model pairs including Models 2 and 4. The *AIC* indicates that Model 2 is inferior to the other models. **Figure 1** shows scatter plots of the relations between the mean preference ratings and 1st-order entropy (**Figure 1E**) and 2nd-order entropy (**Figure 1F**), respectively. These scatter plots also visualize the good separation of preference ratings based on *shape* alone (clusters of *red* and *blue* dots in **Figure 1**; predicted variance of 74% in Model 4). As described by Bertamini et al. (2016), curved patterns were preferred over angular ones. First-order entropy was higher for the curved stimuli (*median*: 4.47) than for the angular ones (*median*: 4.35; Mann–Whitney test, *U* = 80, *p* < 0.0001). A similar relation was obtained for 2nd-order entropy (*median:* 4.26 and 4.15, respectively; *U* = 674, *p* = 0.0001).

**Table 2:** Results from multiple linear regression analyses of the data from Experiment 1 (Bertamini et al., 2016).

Variable	β_i_	*t*-value	*p*-value
Model 1 *(AIC = 475.6. R*^2^_adj_ = 0.75; *F*[5,174] = 106.9; *p* < 0.0001)			

**Shape**	0.70	8.24	<0.0001
lst-order entropy	0.128	1.18	0.239
2nd-order entropy	0.032	0.35	0.725
Edge density	0.072	1.07	0.288
Self-similarity	−0.0007	−0.017	0.986

Model *2 (AIC* = 564.0; *R^2^*_adj_ = 0.58; *F*[2,177] = 124.9; *p* < 0.0001)^1^			

**1st-order entropy**	0.56	7.16	<0.0001
**2nd-order entropy**	0.24	3.10	0.002

Model 3 *(AIC* = 472.8; *R*^2^_adj_ = 0.75; *F*[3,176] = 178.7; *p* < 0.0001)			

**Shape**	0.75	10.92	<0.0001
lst-order entropy	0.05	0.60	0.548
2nd-order entropy	0.10	1.65	0.101

Model 4 (*AIC* = 474.1; *R*^2^_adj_ = 0.74; *F*[1,178] = 521.3; *p* < 0.0001)			

**Shape**	0.86	22.83	<0.0001

*Model 1 is the full model. Model 3 describes the effect of shape (curved or angular), 1st-order entropy and 2nd-order entropy on the preferences ratings. The two measures of entropy are the only predictors in Model 2, while shape is the only predictor in Model 4. Variables that are significant predictors when controlling for the remaining variables are highlighted by bold letters. ^1^Different from Model 1 (F[177,174] = 39.97, p < 0.0001) and Model 3 (F[177,176] = 119.34, p < 0.0001).*

### Discussion

Results matched our expectation that 1st-order entropy and 2nd-order entropy of edge orientations are higher in the curved stimuli than in the angular stimuli. In addition, our findings suggest that the two measures of entropy together predict the preference ratings of the stimuli about as well as their curved/angular shape (Bertamini et al., 2016).

## Experiment 2: Ornamental Geometric Patterns

In the previous experiment, stimuli were relatively simple shapes. In Experiment 2, we therefore asked whether the edge-orientation entropies relate to visual preference also for more complex geometric stimuli. To answer this question, we created a series of artificial ornamental patterns, some of which were vaguely reminiscent of decorative art (for examples, see **Figures 2A–F**).

### Methods

With the freely available *Taprats* computer software^1^ (Kaplan, 2000), author M. G. generated a set of artificial ornamental patterns. The size of the images was 500 pixels × 500 pixels. From this set, we arbitrarily selected a subset of 100 patterns that differed widely not only in edge-orientation entropy but also in self-similarity and edge density (for a more detailed description of these measures, see section “General Methods”). For the exemplary images shown in **Figures 2A–F**, the calculated image properties are listed in **Supplementary Table S1**. Thirty-one participants (*mean* 25.8 years, *range* 18–35 years, 12 male) rated the 100 *Taprats* images in the same session as the stimuli in Experiment 6. The order of the two experiments was randomized. We asked participants to rate the patterns according to the three rating terms *interesting, pleasing* and *harmonious* (see section “General Methods”).

### Results

**Table 3** lists the results of the multiple linear regression analysis. **Supplementary Table S1** displays the median values for the image properties and ratings. The full models (Model 1) can explain 13 and 22% of the variance for the rating terms *pleasing* and *interesting*, respectively. The strongest predictors are 2nd-order entropy (positive effect) and self-similarity (negative effect). The same two variables are also predictors for the rating term *harmonious*, but Model 1 has stronger predictive power (43%). For *harmonious*, the effect of the two variables has an inverse pattern, i.e., there is a negative effect for 2nd-order entropy and a positive effect for self-similarity. For *harmonious* only, 1st-order entropy is also a predictor when controlling for the other variables. Here, self-similarity has a stronger predictive power than the edge-orientation entropies (**Table 3**). Restriction of the model to the two measures of entropy (Model 2 in **Table 3**) yields similar results for *pleasing* (10 and 13%, respectively; *R*^2^ difference test, *F*[97,95] = 2.32, *p* = 0.103). However, Model 2 has slightly less predictive power for *interesting* (17 and 22%, respectively) and much less for *harmonious* (13 and 43%, respectively).

**Table 3:** Results from a multiple linear regression analyses (*Taprats* patterns, Experiment 2).

Variable	β_i_	*t*-value	*p*-value
*Pleasing*			

Model 1 (*AIC* = −509.8; *R^2^_ad_*_j_ = 0.13; *F*[4,95] = 4.60; *p* = .0019)			

lst-order entropy	−0.06	−0.553	0.581
**2nd-order entropy**	0.36	3.171	0.0021
Edge density	0.11	1.032	0.305
**Self-similarity**	−0.23	−2.153	0.034

Model 2 (*AIC* = −509.0; *R*^2^_adj_ = 0.10; *F*[2,97] = 6.69*; p* = 0.0019)			

lst-order entropy	−0.13	−1.206	0.231
**2nd-order entropy**	0.40	3.590	0.0005

*Interesting*			

Model 1 (*AIC* = −481.5; *R*^2^_adj_ = 0.22; *F*[4,95] = 7.87; *p* < 0.0001)			

lst-order entropy	−0.106	−0.975	0.33
**2nd-order entropy**	0.45	4.221	<0.0001
Edge density	0.14	1.347	0.181
**Self-similarity**	−0.28	−2.769	0.0068

Model 2 (*AIC* = −477.7; *R*^2^_adj_ = 0.17; *F*[2,97] = 11.24; *p* < 0.000 1)^1^			

lst-order entropy	−0.19	−1.800	0.075
**2nd-order entropy**	0.50	4.695	<0.0001

*Harmonus*			

Model 1 (*AIC* = −491.7; *R*^2^_adj_ = 0.43; *F*[4,95] = 19.39; *p* < 0.0001)			

**1st-order entropy**	0.24	2.557	0.0121
**2nd-order entropy**	−0.32	−3.544	0.0006
Edge density	−0.11	−1.303	0.196
**Self-similarity**	0.60	6.903	<0.0001

Model 2 (*AIC* = −453.38; *R*^2^_adj_ = 0.13; *F*[2,97] = 8.631; *p* = 0.0004)^2^			

**1st-order entropy**	0.39	3.595	0.0005
**2nd-order entropy**	−0.39	−3.616	0.0005

*^1,2^Different from Model 1: ^1^F[97,95] = 3.84, p = 0.025; and ^2^F[97,95] = 25.74, p < 0.0001. In Model 1, 1st-order entropy, 2nd-order entropy, edge density and self-similarity were predictors of the three aesthetic ratings (pleasing, interesting, and harmonious). Model 2 was restricted to the two measures of entropy. Variables that are significant predictors when controlling for the remaining variables are highlighted by bold letters.*

**Figure 2** displays scatter plots of the relation between some of the variables and the three rating terms. Note that, for 1st-order entropy, values are closer to the maximum value of about 4.585 (for orientation histograms with 24 bins; **Supplementary Table S1**) than for 2nd-order entropy.

### Discussion

Experiment 2 shows that the four calculated variables predict the aesthetic ratings not only for simple shapes (Experiment 1) but also for more complex ornamental patterns. However, the strength of the effect depends on the type of aesthetic rating. Compared to *pleasing* and *interesting*, the effect is stronger for *harmonious*, where self-similarity is a stronger predictor than the two measures of entropy. Moreover, the sign of the effect is opposite for *harmonious* for all four variables.

## Experiment 3: Building Facades

Next, we asked whether edge-orientation entropy is a predictor of aesthetic ratings for objects that we encounter in our everyday environment. Modern humans spend most of their time inside buildings or outside in an urban environment and are thus exposed to a large spectrum of man-made objects, patterns and scenes. It has been proposed that the statistics of the visual environment can have an effect on our psychological well-being (Joye, 2007). The question of what objective characteristics human prefer in their visual environment is thus of considerable interest for architecture and urban development. Therefore, as examples of architecture, we studied images of building facades (Experiment 3) and indoor architectural scenes (Experiment 4).

The majority of people in industrialized countries live in cities. The facades of buildings are among the visual stimuli that are frequently and regularly viewed by a large number of people. Facades are dominated by regularly arranged windows with a structure of mostly cardinal (horizontal and vertical) orientations. This basic pattern can be embellished by the addition of more or less elaborate ornaments and decorations, which can render the facades more pleasant or interesting to look at. Consequently, building facades are well suited to study general visual preferences.

### Methods

**FIGURE 3:**
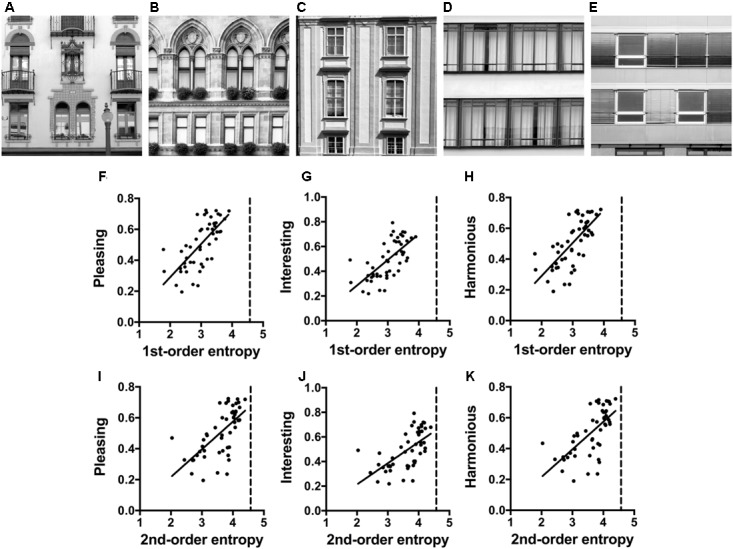
**(A–E)** Examples of the facade photographs (Experiment 3). The values for the image properties calculated for each image are listed in **Supplementary Table S2**. **(F–K)** Dot plots of 1st-order entropy **(F–H)** and 2nd-order entropy **(I–K)** *versus* the aesthetic ratings, averaged across participants. Each dot represents the results for one image. The straight lines indicate the results from simple regression analyses. Significant regressions only are plotted (**F**, *R*^2^ = 0.488; *F*[1,48] = 45.74, *p* < 0.0001; **G**, *R*^2^ = 0.450; *F*[1,48] = 39.19, *p* < 0.0001; **H**, *R*^2^ = 0.493; *F*[1,48] = 46.71, *p* < 0.0001; **I**, *R*^2^ = 0.419; *F*[1,48] = 34.58, *p* < 0.0001; **J**, *R*^2^ = 0.376; *F*[1,48] = 28.92, *p* < 0.0001; **K**, *R*^2^ = 0.418; *F*[1,48] = 34.50, *p* < 0.0001).

Front facades were photographed by author C. R., mostly in the cities of Vienna and Berlin. Images were taken with a digital camera (EOS 500D with EF-S15-85 mm f/3.5-5.6 IS USM lens; Cannon, Tokyo, Japan) and were saved in RAW format. Out of the 175 photographs in the original dataset (Braun et al., 2013), 50 images that covered a variety of decorations were selected for the rating experiment. Each photograph was cropped to select a photographic detail that showed two stories of a building. For monochrome versions of the images, we used the L channel of the Lab color space, in which channels are weighted according to their perceived luminosity, similar to the luma transform, which was mentioned in the section “General Methods, Entropy of Edge Orientations”. The image size was 500 pixels × 500 pixels. The luminance histograms were equalized such that they shared the same luminance distribution, which was computed as the mean cumulative histogram of all images. Examples are shown in **Figures 3A–E** and their image properties are listed in **Supplementary Table S2**.

The group of participants was different from the group that participated in Experiment 2. Twenty-seven participants (mean age: 26.4 years, range: 20–40 years, 14 male) were asked to evaluate 50 facade photographs according to the three rating terms *interesting, pleasing*, and *harmonious* (see section “General Methods”) in the same session as the stimuli in Experiment 5. The order of the experiments and the three ratings was randomized.

### Results

The results of the multiple linear regression analysis are listed in **Table 4**. The full models with all four variables (Model 1) explain 55, 51, and 29% of the variance in the ratings for *interesting, pleasing*, and *harmonious*, respectively. For *pleasing* and *interesting*, the strongest predictor was 1st-order entropy whereas the four variables were similarly strong predictors for *harmonious*. Restriction of the model to the two measures of entropy (Model 2 in **Table 4**) yields results similar to Model 1 for *interesting* (48 and 51%, respectively; *R*^2^ difference test, *F*[47,45] = 2.67, *p* = 0.081), but Model 2 has less predictive power for *pleasing* (48 and 55%, respectively) and for *harmonious* (21 and 29%, respectively). For all rating terms, 1st-order entropy is a strong positive predictor whereas 2nd-order entropy has a negative effect. **Figures 3F–K** shows scatter plots for the two measures of entropy with the results from single linear regression. For *pleasing*, *interesting*, and *harmonious*, 1st-order entropy alone explains 49, 45, and 42%, and 2nd-order entropy alone 42, 38, and 42% of the variance, respectively.

**Table 4:** Results from a multiple linear regression analyses for facade photographs (Experiment 3).

Variable	β_i_	*t*-value	*p*-value
*Pleasing*			

Model 1 (*AIC* = −224.0;*R*^2^_adj_ = 0.55; *F*[4,45] = 16.11; *p* < 0.0001)			

lst-order entropy	0.72	1.891	0.065
2nd-order entropy	−0.08	−0.222	0.823
Edge density	0.21	1.851	0.071
Self-similarity	0.16	1.581	0.138

Model 2 (*AIC* = −218.85; *R*^2^_adj_ = 0.48; *F*[2,47] = 16.11; *p* < 0.0001)^1^			

**1st-order entropy**	1.11	2.890	0.0058
2nd-order entropy	−0.42	−1.101	0.276

*Interesting*			

Model 1 (*AIC* = −215.7; *R*^2^_adj_ = 0.51; *F*[4,45] = 13.99; *p* < 0.0001)			

**1st-order entropy**	0.88	2.209	0.032
2nd-order entropy	−0.24	−0.602	0.550
Edge density	0.16	1.323	0.192
Self-similarity	0.14	1.258	0.215

Model 2 (*AIC* = −214.1; *R*^2^_adj_ = 0.48; *F*[2,47] = 23.64; *p* < 0.0001)			

**1st-order entropy**	1.18	3.067	0.0036
2nd-order entropy	−0.51	−1.313	0.196

*Harmonius*			

Model 1 (*AIC* = −221.9; *R*^2^_adj_ = 0.29; *F*[4,45] = 6.02; *p* = 0.0006)			

lst-order entropy	0.20	0.408	0.685
2nd-order entropy	0.26	0.541	0.591
Edge density	0.21	1.449	0.154
Self-similarity	0.20	1.473	0.148

Model 2 (AIC = −218.8; *R*^2^_adj_ = 0.21; *F*[2,47] = 7.82; *p* = 0.0012)^2^			

lst-order entropy	0.61	1.288	0.204
2nd-order entropy	−0.12	−0.243	0.809

*^1,2^Different from Model 1: ^1^F[47,45] = 4.54, p = 0.016; and ^2^F[47,45] = 3.42, p = 0.042. In Model 1, 1st-order entropy, 2nd-order entropy, edge density and self-similarity were predictors of the three aesthetic ratings (pleasing, interesting, and harmonious). Model 2 was restricted to the two measures of entropy. Variables that are significant predictors when controlling for the remaining variables are highlighted by bold letters.*

### Discussion

Results for the facades suggest that edge-orientation entropy plays a role also in the aesthetic rating of man-made objects. For all three rating terms, edge-orientation entropy is a strong predictor. Facades are rated as more *interesting* and *pleasing* when overall edge orientation is more evenly distributed across the full spectrum of orientations. This effect is possibly achieved by complementing the cardinal orientations, which are prevalent in the window frames, by various ornaments and decorations. When oblique orientations are introduced, edges are more uniformly distributed across orientations (higher 1st-order entropy). Moreover, for *harmonious*, higher ratings are obtained when the spatial distribution of edge orientations across the facades is more irregular (higher 2nd-order entropy; for an example, see **Figure 3A**).

## Experiment 4: Interior Scenes

**FIGURE 4:**
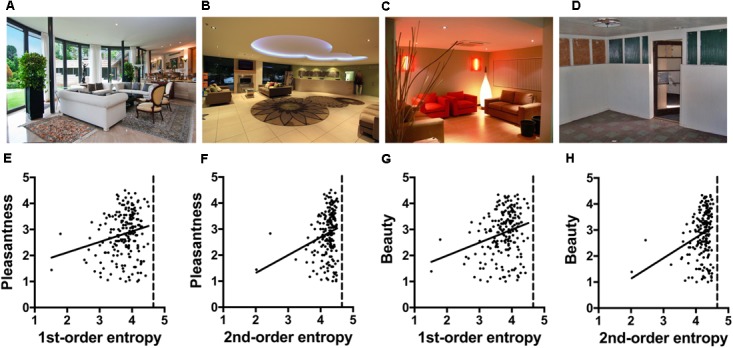
**(A–D)** Examples of the interior scene photographs (Vartanian et al., 2013; Experiment 4). The values for the image properties calculated for each image are listed in **Supplementary Table S3**. **(E–H)** Dot plots of 1st-order entropy **(E,G)** and 2nd-order entropy **(F,H)** *versus* the ratings for *pleasantness* **(E,F)** and *beauty* **(G,H)**, averaged across participants. Each dot represents the results for one image. The straight lines indicate the results from simple regression analyses (**E**, *R*^2^ = 0.043; *F*[1,198] = 8.99, *p* = 0.0031; **F**, *R*^2^ = 0.053; *F*[1,198] = 11.17, *p* = 0.0010; **G**, *R*^2^ = 0.065; *F*[1,198] = 13.68, *p* = 0.0003; **H**, *R*^2^ = 0.070; *F*[1,198] = 14.80, *p* = 0.0002). The images in **(A–D)** were reproduced with kind permission from Dr. O. Vartanian.

Urban dwellers worldwide spend most of their time indoors. Therefore, as another example of man-made stimuli of general and widespread appearance, we analyzed 200 images of modern interior architecture. The images were previously studied by Vartanian et al. (2013) and varied in several architectural aspects, including their degree of curvilinearity (for examples, see **Figures 4A–D**). Based on results from approach-avoidance decisions and fMRI data, Vartanian et al. (2013) concluded that the well-established preference for curvilinear objects extends to architecture. In the present *post hoc* analysis, we asked whether the image properties calculated for these images relate to the ratings of *pleasantness* and *beauty* ratings that were obtained in the study by Vartanian et al. (2013).

### Methods

The 200 color images of interior scenes from the study of Vartanian et al. (2013) were kindly provided by Oshin Vartanian, University of Toronto. In their study, the authors had these stimuli rated by eighteen participants for *pleasantness* and *beauty* on a five-point scale with anchors “very unpleasant”/“very pleasant” and “very ugly”/“very beautiful,” respectively, after the completion of the fMRI scanning in their study. Note that the two ratings terms are not independent of each other. The *pleasant* ratings accounted for 58% of the observed variance in *beautiful* ratings. The images were systematically controlled for the architectural features *contour* (curvilinear or rectilinear), *ceiling height* (high or low) and *openness of space* (open or closed; Vartanian et al., 2013). In the present study, we used these three binary variables alongside the four measured image properties (for mean values, see **Supplementary Table S3**) as independent variables in a multiple linear regression analysis to predict the ratings of *pleasantness* and *beauty*, respectively, which had been obtained by Vartanian et al. (2013) (Model 1). *Contour* (curvilinear or rectilinear) served as a binary variable to indicate curvilinearity (**Table 1**). We also ran the same analysis with the two measures of entropy only (Model 2).

### Results

The full model (Model 1), which includes the four image properties and the three architectural control variables, explains 14 and 15% of the variance for the *pleasantness* and *beauty* ratings, respectively (**Table 5**). For both ratings, *openness* but not *contour* or *ceiling height* are predictors when the other variables are controlled for. Moreover, edge density and self-similarity are also predictors. The restricted Model 2 has less predictive power than Model 1, and predicted 4 and 6% of the variance, respectively. *Contour* alone did not predict the ratings. The scatter plots in **Figures 4E–H** visualize the minor, positive effect of the two edge-orientation entropies on the ratings.

**Table 5:** Results from a multiple linear regression analyses for interior scene photographs (Experiment 4).

Variable	β_i_	*t*-value	*p*-value
*Pleasantness*			

Model 1 (*AIC* = −80.98; *R*^2^_adj_ = 0.14; *F*[7,192] = 5.55; *p* < 0.0001)			

lst-order entropy	0.12	0.797	0.426
2nd-order entropy	0.13	0.870	0.385
**Edge density**	0.24	3.243	0.0014
**Self-similarity**	−0.18	−2.580	0.0106
Contour	−0.005	−0.072	0.943
**Openness**	0.20	2.897	0.0042
Ceiling height	0.025	0.371	0.0711

Model 2 (*AIC* = −65.1; *R*^2^_adj_ = 0.044; *F*[2,197] = 5.57; *p* < 0.0045)^1^			

lst-order entropy	0.017	0.113	0.910
2nd-order entropy	0.22	1.447	0.149

*Beauty*			

Model 1 (*AIC* = −79.53; *R*^2^_adj_ = 0.15; *F*[7,192] = 5.82; *p* < 0.0001)			

lst-order entropy	0.17	1.163	0.246
2nd-order entropy	0.11	0.766	0.444
**Edge density**	0.22	3.061	0.0025
**Self-similarity**	−0.18	−2.544	0.0117
Contour	0.045	0.679	0.498
**Openness**	0.18	2.619	0.0095
Ceiling height	0.037	0.539	0.591

Model 2 *(AIC* = −65.88; *R*^2^_adj_ = 0.062; *F*[2,197] = 7.59; *p* = 0.0007)^2^			

lst-order entropy	0.16	0.647	0.518
2nd-order entropy	0.27	1.209	0.228

*^1,2^Different from Model 1: ^1^F[197,192] = 5.31, p = 0.00014; and ^2^F[197,192] = 4.82, p = 0.00036. In Model 1, 1st-order entropy, 2nd-order entropy, edge density, self-similarity and the three architectural variables contour, openness, and ceiling height were predictors for the three types of aesthetic ratings (pleasantness and beauty). Model 2 was restricted to the two measures of entropy. Variables that are significant predictors when controlling for the remaining variables are highlighted by bold letters.*

### Discussion

Vartanian et al. (2013) reported that participants were more likely to judge interior spaces as beautiful if they were curvilinear as opposed to rectilinear. In their fMRI experiment, beauty ratings co-varied with activation of a brain network that is known to underlie the aesthetic evaluation of different types of visual stimuli. The present *post hoc* analysis of the post-scanning evaluations of the same stimuli, reveal that *openness* is a much stronger predictor of the ratings of *pleasantness* and *beauty* than *contour* or *ceiling height*. Moreover, 1st-order and 2nd-order entropy alone also predict some of the variance of the ratings of *pleasantness* and *beauty* (about 4 and 6%, respectively; **Table 5**). Compared to the building facades (**Figures 3F–K**), the values for the entropies are relatively high and, for 2nd-order entropy, close to the maximum value of about 4.585 (dashed vertical lines in **Figures 4E–H**). However, the respective linear correlations with the ratings (**Figures 4E–H**) are relatively weak.

## Experiment 5: Music Album Covers

Following artificial patterns and veridical photographs of architecture, we next investigated the role of edge-orientation entropies in the aesthetic rating of music album covers. It has been shown that low-level image features of music-related visual information, such a cover art, can differ between music genres (Libeks and Turnbull, 2011). To analyze a spectrum of music album covers, we studied covers from three different music genres, which were classified according to the MUSIC model by Rentfrow et al. (2011). This model describes musical preference in relation to sonic and psychological attributes in various genres and contains five different labels: *Mellow* (relaxing, quiet sometimes sad music such as R&B and soft rock), *Unpretentious* (country, folk and singer/songwriter genres), *Sophisticated* (jazz and other inspiring, dynamic music), *Intense* (distorted, loud aggressive music, such as classic rock, punk and heavy metal) and *Contemporary* (electric, pop genres). In the present study, we arbitrarily chose pop (short for popular music) as an example of *Contemporary* music, metal for *Intense* music and classic music for *Sophisticated* music.

### Methods

**FIGURE 5:**
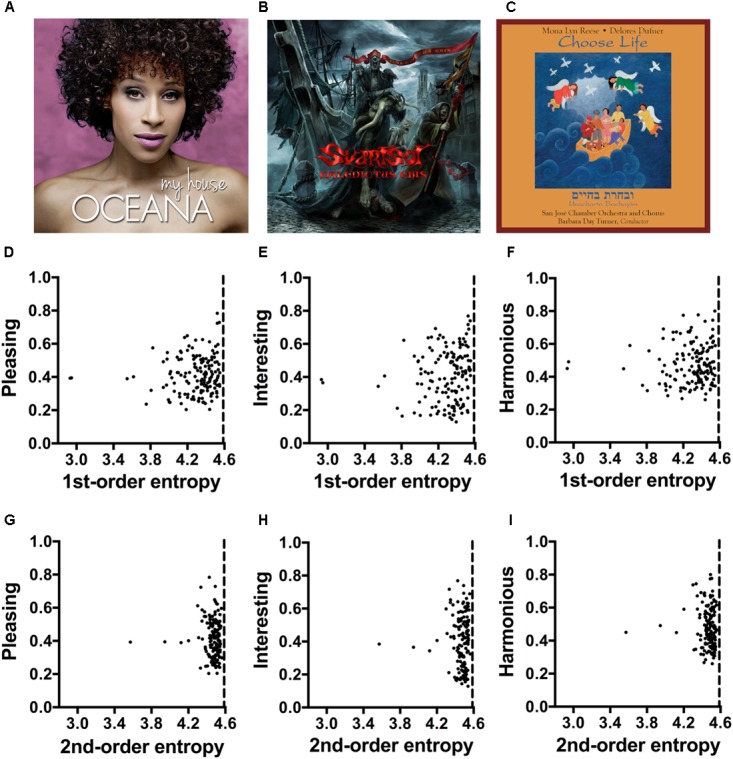
**(A–C)** Typical examples of album covers (Experiment 5) for pop music **(A)**, metal music **(B)** and classic music **(C)**. The values for the image properties of each image are listed in **Supplementary Table S4**. Due to copyright issues, the covers used in the rating experiment are not reproduced here. The images in **(A–C)** are in the public domain and were downloaded from Wikimedia Commons. **(D–I)** Dot plots of 1st-order entropy **(D–F)** and 2nd-order entropy **(G–I)** *versus* the ratings for *pleasing* **(D,G)**, interesting **(E,H)** and harmonious **(F,I)**, averaged across participants. Each dot represents the results for one image. Single regression analyses did not yield slopes that were significantly different from zero (**D**, *R*^2^ = 0.009; *F*[1,148] = 1.31, *p* = 0.254; **E**, *R*^2^ = 0.013; *F*[1,148] = 1.92, *p* = 0.168; **F**, *R*^2^ = 0.002; *F*[1,148] = 0.358, *p* = 0.551; **G**, *R*^2^ = 0.003; *F*[1,148] = 0.41, *p* = 0.524; **H**, *R*^2^ < 0.01; *F*[1,148] = 0.001, *p* = 0.975; **I**, *R*^2^ = 0.007; *F*[1,148] = 1.06, *p* = 0.305).

For each music genre (metal, pop and classic music), we collected 50 images (150 images in total) from private music collections and the internet (for examples, see **Figures 5A–C**). The 150 images were presented as a block in randomized order. The same 27 participants as in Experiment 3 took part in the experiment. The order of the two experiments was randomized.

For multiple linear regression, we used a model in which all images were analyzed together, and the four image properties and the three music genres were independent variables (Model 1; **Table 6**). With this model, we also analyzed the music genres, which were represented by a categorical variable. In addition, Model 2 assessed the effect of the entropies alone.

**Table 6:** Results from a multiple linear regression analyses for music album covers (Experiment 5).

Variable	β_i_	*t*-value	*p*-value
*Pleasing*			

Model 1 (*AIC* = −653.5: *R*^2^_adj_ = 0.045; *F*[6,143] = 2.17; *p* = 0.049)			

**1st-order entropy**	0.38	2.376	0.0188
**2nd-order entropy**	−0.370	−2.371	0.0191
Edge density	0.042	0.463	0.644
Self-similarity	0.023	0.259	0.796
Genre metal	0.282	1.629	0.105
Genre pop	0.031	0.593	0.554

Model 2 (*AIC* = −657.4; *R*^2^_adj_ = 0.045; *F*[2,147] = 4.54; *p* = 0.0122)			

**1st-order entropy**	0.41	2.942	0.0038
**2nd-order entropy**	−0.38	−2.777	0.0062

*Interesting*			

Model 1 (*AIC* = −593.3: *R*^2^_adj_ = 0.25; *F*[6,143] = 9.47; *p* < 0.0001)			

lst-order entropy	0.268	1.919	0.057
2nd-order entropy	−0.253	−1.839	0.068
Edge density	0.132	1.639	0.103
Self-similarity	0.092	1.151	0.252
**Genre metal**	0.833	5.448	<0.0001
Genre pop	0.060	1.319	0.189

Model 2 (*AIC* = −556.7; *R*^2^_adj_ = 0.024; *F*[2,147] = 11.24; *p* < 0.0640)^1^			

**1st-order entropy**	0.33	2.366	0.0193
2nd-order entropy	−0.267	−1.910	0.0581

*Harmonious*			

Model 1 (*AIC* = −645.9; *R*^2^_adj_ = 0.093; *F*[6,143] = 3.55; *p* = 0.0026)			

**1st-order entropy**	0.39	2.511	0.0131
**2nd-order entropy**	−0.37	−2.458	0.0152
Edge density	−0.18	−1.969	0.0509
Self-similarity	0.014	0.159	0.874
**Genre metal**	−0.37	−2.182	0.0308
**Genre pop**	−0.12	−2.440	0.0159

Model 2 (*AIC* = −640.5; *R*^2^_adj_ = 0.035; *F*[2,147] = 3.74; *p* = 0.026)			

**1st-order entropy**	0.35	2.536	0.0126
**2nd-order entropy**	−0.37	−2.666	0.0085

*^1,2^Different from Model 1: ^1^F[143,147] = 12.38, p < 0.0001; and ^2^F[143,147] = 3.331, p = 0.0121. Model 1 comprised all four image properties as well as music genre to predict the aesthetic ratings. Model 2 was restricted to 1st-order entropy and 2nd-order entropy. Variables that are significant predictors when controlling for the remaining variables are highlighted by bold letters.*

### Results

The mean values for 1st-order entropy and 2nd-order entropy are close to the theoretical maximum values (about 4.585; **Supplementary Table S4**; see dashed vertical lines in **Figures 5D–I**). **Table 6** lists the results from multiple linear regression for the full model, which includes all four image properties and music genre (Model 1), calculated on the basis of all 150 images. Model 1 explains 4.5, 25, and 9.3% of the rating variability for *pleasing*, *interesting*, and *harmonious*, respectively. In this model, larger 1st-order entropy tends to increase ratings and larger 2nd-order entropy tends to decrease ratings for each of the three rating terms (**Table 6**). However, single linear regressions did not yield slopes significantly different from zero for the two measures of entropy (**Figures 5D–I**). For all three rating terms, music genre contributes substantially to the predictions of the model. For *interesting*, the music genre *metal* is by far the strongest predictor. For *harmonious*, the variables *pop* and *metal* are significant predictors when the other variables are controlled for.

When the model is restricted to the two measures of entropy (Model 2), predictive power does not decrease for *pleasing* (*R*^2^ difference test, *F*[143,147] = 0.984, *p* = 0.419). However, the *AIC* suggests that Model 2 is better than Model 1. Compared to Model 1, Model 2 does not allow a significant prediction for *interesting*, and predicts only 3.5% of the variance for *harmonious*.

When music genres are analyzed separately (results not shown in a table), the two models do not predict the ratings for *classic* and *metal* covers. However, self-similarity alone predicts ratings of *classic* album covers for *pleasing* (*R*^2^_adj_ = 0.086, *F*[1,48] = 5.59, *p* = 0.022) and *interesting* (*R*^2^_adj_ = 0138, *F*[1,48] = 8.84, *p* = 0.005), but not for *harmonious*. Model 1 predicts ratings of *pop* album covers for *pleasing* (*R*^2^_adj_ = 0.14, *F*[4,45] = 3.05, *p* = 0.026), *interesting* (*R*^2^_adj_ = 0.13, *F*[4,45] = 2.868, *p* = 0.034) and *harmonious* (*R*^2^_adj_ = 0.27, *F*[4,45] = 5.547, *p* = 0.0010), with 1st-order entropy (positive effect) and 2nd-order entropy (negative effect) being the strongest predictors.

### Discussion

The variance predicted by the full model, which includes the four image properties and music genre as variables, was relatively low for *pleasing* (4.5%; **Table 6**) and *harmonious* (9.3%). For *pleasing*, the two edge-orientation entropies alone (Model 2) accounted for a similar amount of variability (4.5%) but the entropies were rather weak predictors for *harmonious* (3.5% only). The higher predictive power of Model 1 for *interesting* was largely predicted by music genre, specifically the *metal* category (25%), which was rated more *interesting* than the other two music genres on average (**Supplementary Table S4**). As predictors, the image properties play less of a role (**Table 6**). However, the relatively weak influence of the edge-orientation entropies on the ratings may also be due to a ceiling effect because values for both entropies (**Supplementary Table S4**) are closer to their maximum value than in Experiments 2–4 (**Supplementary Tables S1**–**S3**). More of the variances in the ratings is explained if the music genres are analyzed separately, at least for *classic* and *pop* covers. Here, Model 1 and self-similarity, respectively, account for 8–27% of the variances within the two music genres.

## Experiment 6: Angular/Curved Line Patterns

Experiment 6 is different from the previous set of experiments. In this second part of our study, we created artificial patterns that were composed of either curved lines or straight lines that formed sharp angles. The aim was to directly manipulate entropy and curvature. Each stimulus consisted of 40 separate line elements and was thus considerably more complex than the stimuli analyzed in Experiment 1. More importantly, we systematically increased and decreased the 2nd-order edge-orientation entropy by manipulating the layout of the lines using an evolutionary approach that changed a stimulus stepwise until a desired entropy value was reached. In this way, we obtained stimuli that varied in their curvilinearity or their 2nd-order edge-orientation entropy, but were composed of comparable sets of lines. This allows us, for the first time, to study the relative contributions of curvilinearity and edge-orientation entropy to the aesthetic ratings. Like in Experiments 2, 3, and 5, we asked participants to rate the stimuli according to how *pleasing*, *interesting*, and *harmonious* they were perceived. Based on the postulated preference of humans for curvilinear stimuli, we expected that the curved stimuli would be rated more highly than the angular patterns. Moreover, because artworks possess high edge-orientation entropy (Redies et al., 2017), we wondered whether the stimuli with high 2nd-order entropy would be rated more highly than the stimuli with low 2nd-order entropy.

### Methods

#### Generation of the Stimuli

**FIGURE 6:**
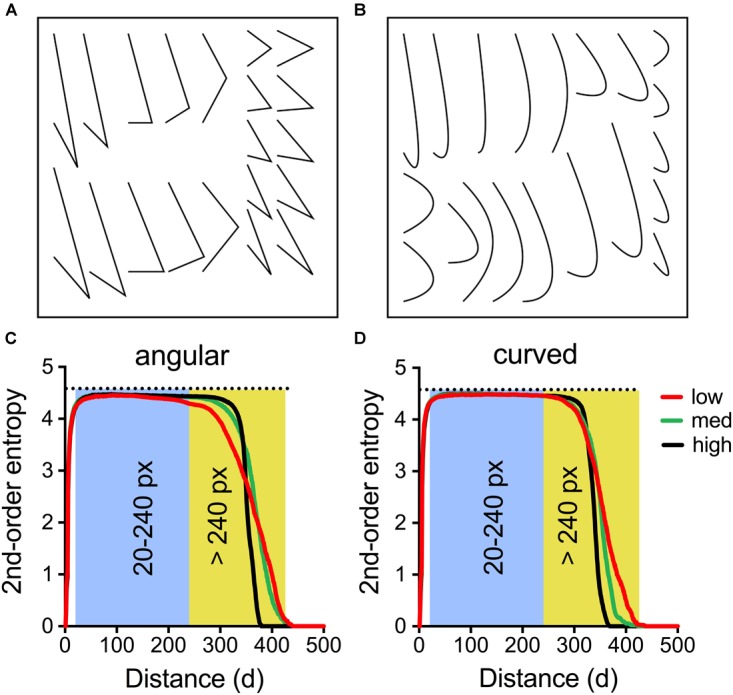
Characteristics of the line stimuli used in Experiment 6. The stimuli were composed of a set of either angular line elements **(A)** or curved ones **(B)**. Each set was used twice in each stimulus, resulting in 40 line elements per stimulus (see **Figure 7**). In **(C,D)**, the mean 2nd-order entropy for the angular stimuli **(C)** and the curved stimuli **(D)** was plotted as a function of pixel distance for the 10 stimuli with lowest entropy values above 240 pixels distance (*low*, *red* lines; angular *Median*: 3.25, *Range*: 3.19–3.44; curved *Median*: 3.44, *Range*: 3.33–3.52), with the values closest to the median entropy (*med*, *green* lines; angular *Median*: 3.77, *Range*: 3.68–3.83; curved *Median*: 3.80, *Range*: 3.70–3.90) and with the highest entropy values (*high*, *black* lines; angular *Median*: 4.11, *Range*: 4.06–4.20; curved *Median*: 4.14, *Range*: 4.09–4.21), respectively. The shaded areas indicate the distance between 20–240 pixels and >240 pixels, respectively.

**FIGURE 7:**
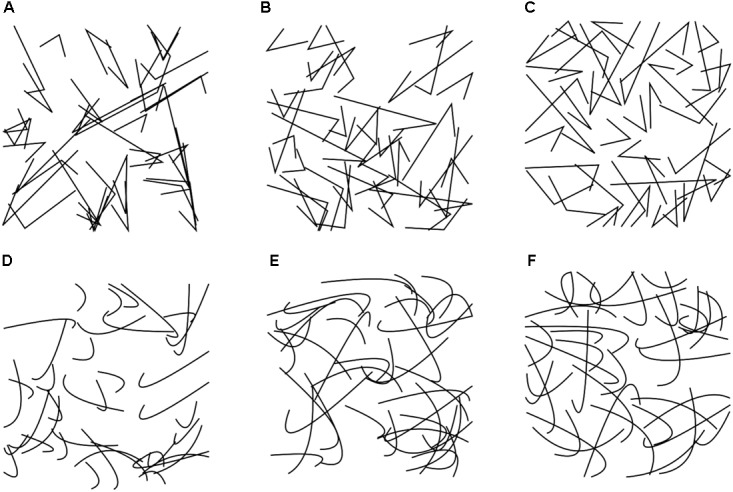
Typical examples of the angular **(A–C)** and curved **(D–F)** stimuli (Experiment 6). The values for the image properties of each image are listed in **Supplementary Table S5**. Second-order entropy in the images is low **(A,D)**, intermediate **(B,E)**, and high **(C,F)**, respectively.

We created images within specific ranges of entropy using an evolutionary procedure, which will be described in the following. At the outset, we defined a set of fixed line elements, each consisting of three points in the plane. The points for each element can be connected by drawing a line from the first to the second point and from the second to the third one. The result is a triangle with one open side because there is no connection from the first to the last point (**Figure 6A**). Alternatively, the three points can be expressed as a quadratic Bézier curve. The resulting element has the same start and end points as the first one, but no sharp corner (**Figure 6B**). We refer to the two types of lines as “angular” and “curved,” respectively (for examples, see **Figure 7**). As shown in **Figures 6A,B**, each set of 20 line elements contained shorter and longer lines with different relative positions of the intervening points. Two sets of the 20 line elements shown in **Figure 6** were used for each stimulus, resulting in a total of 40 elements.

By an evolutionary procedure, we generated stimuli with a wide range of 2nd-order edge-orientation entropy. This was accomplished by manipulating the position and orientation of each stimulus until it reached a desired entropy value. The procedure to achieve this goal was as follows: In a first step, we set each line element to a random position on the canvas and measured the 2nd-order edge-orientation entropy of the resulting image containing all elements. A mutation of this seed image was then obtained by altering the position of each of its elements with probability *p* = 0.1 by either translation or rotation, either of which was selected randomly. In case of a translation, an element was shifted in a random direction by a random distance between zero and the size of the canvas times a strength factor. We started with a strength factor of 0.5 and decreased it linearly until it reached 0.01, so that changes became successively smaller at later stages of the process. In case of a rotation, we rotated an element around its own center by an angle between −π/2 and π/2 times the same strength factor. Whenever parts of an element had been shifted to positions outside of the canvas after mutation, they were shifted back.

After each mutation, we measured the total 2nd-order entropy of the resulting image for all pairs of edges that were separated by more than 20 pixels, including pairs of edges that were separated by more than 240 pixels. If their entropy value was closer to the desired value, we used the mutated image as the new seed image for a future generation in the evolutionary procedure. If the entropy value was farther, the image was discarded and the procedure started anew with the seed image. In this way, a series of images was generated with entropy values that converged on the desired value; the procedure was stopped when an image with a value close to the desired value was generated. For technical reasons, the resulting images had to be transformed to a different image format for a better display of the lines in the rating experiment. With this procedure, we obtained 50 images each with angular and curved lines, respectively (100 images in total), which covered a wide range of 2nd-order entropy values (for examples, see **Figure 7**).

#### Rating Experiment

Rating was carried out in the same session as Experiment 2. The order of the two experiments was randomized. Like in Experiment 2, the size of the images was 500 pixels × 500 pixels. Again, we asked participants how *interesting, pleasant* and *harmonious* they rated the stimuli (see section “General Methods”). Participants were the same 31 persons who took part in Experiment 2. The order of the two experiments was randomized. In the multiple linear regression, *Shape* (curved or angular) served as a binary variable to indicate curvilinearity (**Table 1**).

### Results

**FIGURE 8:**
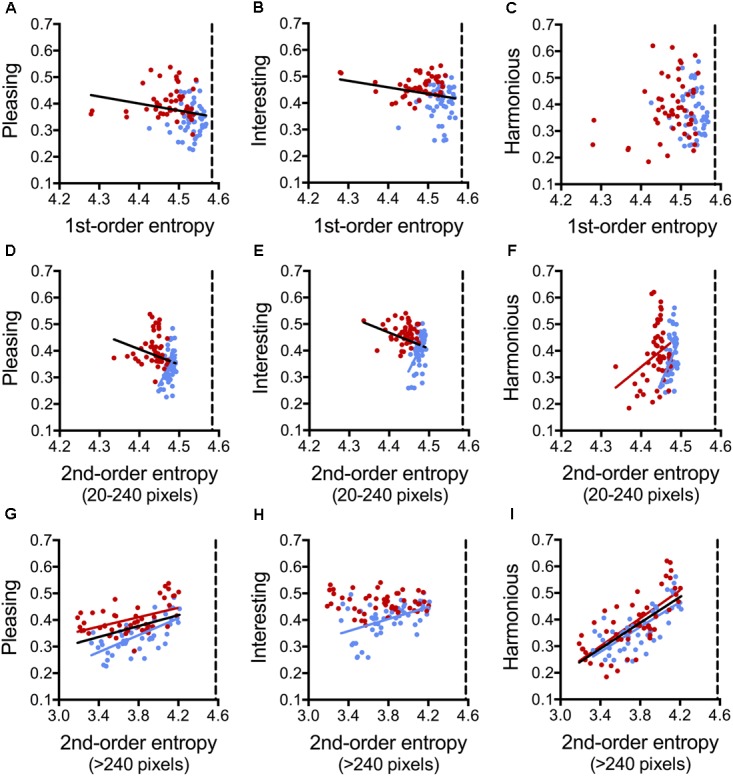
Dot plots of 1st-order entropy **(A–C)**, 2nd-order entropy (20–240 pixels distance; **D–F**), and 2nd-order entropy (>240 pixels distance; **G–I**) *versus* the ratings for *pleasing* **(A,D,G)**, *interesting* **(B,E,H)**, and *harmonious* **(C,F,I)**, averaged across participants (Experiment 6). Each dot represents the results for one image (*red*, angular patterns; and *blue*, curved patterns). Note that the scaling of the x-axis is different in **(A–F)** and **(G–I)**. The straight lines indicate the results from simple regression analyses (**A**, both, angular and curved [*black* line]: *R*^2^ = 0.045; *F*[1,198] = 4.64, *p* = 0.013; **B**, both [*black* line]: *R*^2^ = 0.049; *F*[1,198] = 5.07, *p* = 0.034; **D**, both [*black* line]: *R*^2^ = 0.045; *F*[1,198] = 4.64, *p* = 0.034; curved [*blue* line]: *R*^2^ = 0.201; *F*[1,48] = 12.08, *p* = 0.0011; **E**, both [*black* line]: *R*^2^ = 0.049; *F*[1,198] = 5.07, *p* = 0.027; curved [*blue* line]: *R*^2^ = 0.246; *F*[1,48] = 15.64, *p* = 0.0003; **F**, angular [*red* line]: *R*^2^ = 0.092; *F*[1,48] = 4.86, *p* < 0.032; curved [*blue* line]: *R*^2^ = 0.189; *F*[1,48] = 11.15, *p* < 0.0016; **G**, both [*black* line]: *R*^2^ = 0.190; *F*[1,98] = 22.91, *p* < 0.0001; angular [*red* line]: *R*^2^ = 0.219; *F*[1,48] = 13.48, *p* = 0.0006; curved [*blue* line]: *R*^2^ = 0.460; *F*[1,48] = 40.92, *p* < 0.0001; **H**, curved [*blue* line]: *R*^2^ = 0.227; *F*[1,48] = 14.12, *p* = 0.0005; **I**, both [*black* line]: *R*^2^ = 0.499; *F*[1,98] = 97.68, *p* < 0.0001; angular [*red* line]: *R*^2^ = 0.521; *F*[1,48] = 52.19, *p* < 0.0001; curved [*blue* line]: *R*^2^ = 0.533; *F*[1,48] = 54.84, *p* < 0.0001).

For the 100 images used in the rating experiment, we calculated 1st-order entropy, 2nd-order entropy (separately for the distance ranges of 20–240 pixels and >240 pixels, respectively), edge density and self-similarity (**Supplementary Table S5**). In addition, we plotted the dependence of 2nd-order entropy on pixel distance (for angular stimuli, see **Figure 6C**; for curved stimuli, see **Figure 6D**). In both plots, 2nd-order entropy values are plotted as the means for the 10 stimuli with the highest values (*black* lines), the 10 stimuli with the values closest to the median value (*green* lines) and the 10 stimuli with the lowest values (*red* lines), respectively. For both angular and curved stimuli, large absolute differences in 2nd-order entropy were observed for distances of >240 pixel distance, whereas 2nd-order entropy for shorter distance (20–240 pixels) and 1st-order entropy assumed values close to the maximum value (about 4.585; horizontal dotted line in **Figures 6C,D**; see also **Figure 8**). Note that the largest edge pair distances in the stimuli are about 350–400 pixels (**Figures 7C,D**). Therefore, the variability in the 2nd-order entropy, which was generated during the evolutionary procedure, is mainly driven by edge pairs that are spaced more widely apart. Moreover, for the stimuli with maximized 2nd-order entropy (*black* lines in **Figures 6C,D**), high entropy values were maintained with increasing distances, to decrease sharply for the most distant edge pairs. In contrast, 2nd-order entropy decreased more gradually in the stimuli, in which 2nd-order entropy was minimized (*red* lines in **Figures 6C,D**).

On average, the angular stimuli had lower 1st-order entropy values and lower 2nd-order entropy values for the range of 20–240 pixel distance. They were rated as more *pleasing* and more *interesting* than the curved stimuli on average (**Supplementary Table S5**). The other measures did not differ significantly between angular and curved stimuli.

The three ratings differed in the variables that had the strongest effect on the ratings (**Table 7**). For *pleasing*, the full Model 1 explained 63% of the variance in the rating. The model with the variable *shape* (Model 4) accounted for about half as much the variability as Model 2, which comprised the three entropy variables only (21% versus 42%, respectively). The effect of 2nd-order entropy on the rating was negative for shorter distances (20–240 pixels) and positive for larger distances (>240 pixels).

**Table 7:** Results from multiple linear regression analyses of the data from Experiment 6.

Variable	β_i_	*t*-value	*p*-value
*Pleasing*			

Model 1 (*AIC* = −637.4; *R*^2^_adj_ = 0.63; *F*[6,93] = 29.41; *p* < 0.0001)			

**Shape (curved)**	−0.27	−2.86	0.0052
1st-order entropy	0.03	0.34	0.734
**2nd-order entropy (20–240 pixels)**	−0.34	−2.91	0.0045
**2nd-order entropy (>240 pixels)**	0.20	2.11	0.038
**Edge density**	0.41	4.90	<0.0001
**Self-similarity**	0.26	3.46	0.0008

Model 2 (*AIC* = −594.6; *R*^2^_adj_ = 0.42; *F*[3,96] = 24.9; *p* < 0.0001)^1^			

1st-order entropy	0.02	0.15	0.883
**2nd-order entropy** (20–240 pixels)	−0.57	−4.81	<0.0001
**2nd-order entropy** (>240 pixels)	0.69	7.99	<0.0001

Model 3 (*AIC* = −604.1; *R*^2^_adj_ = 0.48; *F*[4,95] = 23.61; *p* < 0.0001)^2^			

**Shape (curved)**	−0.36	−3.40	<0.0001
1st-order entropy	0.04	0.39	0.695
**2nd-order entropy (20–240 pixels)**	−0.30	−2.21	0.030
**2nd-order entropy (>240 pixels)**	0.61	7.14	<0.0001

Model 4 (*AIC* = −565.6; *R*^2^_adj_ = 0.21; *F*[l,98] = 27.3; *p* < 0.0001)^3^			

**Shape (curved)**	−0.47	−5.23	<0.0001

*Interesting*			

Model 1 (*AIC* = −638.6; *R*^2^adj = 0.55; *F*[6,93] = 21.05; *p* < 0.0001)			

**Shape (curved)**	−0.33	−3.19	0.002
1st-order entropy	0.04	0.37	0.709
2nd-order entropy (20–240 pixels)	−0.12	−0.93	0.357
**2nd-order entropy (>240 pixels)**	−0.24	−2.30	0.024
Edge density	0.71	7.64	<0.0001
Self-similarity	−0.003	−0.04	0.97

Model 2 (*AIC* = −576.7; *R*^2^_adj_ = 0.14; *F*[3,96] = 6.26; *p* = 0.0006)^4^			

1st-order entropy	0.01	0.043	0.965
**2nd-order entropy (20–240 pixels)**	−0.44	−3.01	0.003
**2nd-order entropy (>240 pixels)**	0.32	3.04	0.003

Model 3 (*AIC* = −593.0; *R*^2^_adj_ = 0.27; *F*[4,95] = 10.35; *p* < 0.0001)^5^			

**Shape (curved)**	−0.54	−4.37	<0.0001
1st-order entropy	0.04	0.35	0.724
2nd-order entropy (20–240 pixels)	−0.03	−0.19	0.848
2nd-order entropy (>240 pixels)	0.20	1.98	0.051

Model 4 (*AIC* = −593.7; *R*^2^_adj_ = 0.26; *F*[l,98] = 35.3; *p* < 0.0001)^6^			

**Shape (curved)**	−0.51	−5.95	<0.0001

*Harmonious*			

Model 1 (*AIC* = −589.2; *R*^2^_adj_ = 0.72; *F*[6,93] = 43.71; *p* < 0.0001)			

Shape (curved)	−0.09	−1.06	0.293
1st-order entropy	0.02	0.28	0.780
2nd-order entropy (20–240 pixels)	−0.12	−1.15	0.251
**2nd-order entropy (>240 pixels)**	0.32	3.90	0.0002
**Edge density**	0.27	3.69	.0004
**Self-similarity**	0.47	7.12	<0.0001

Model 2 (*AIC* = −535.4; *R*^2^_adj_ = 0.51; *F*[3,96] = 35.20; *p* < 0.0001)^7^			

1st-order entropy	0.03	0.29	0.775
2nd-order entropy (20–240 pixels)	−0.20	−1.80	0.075
**2nd-order entropy (>240 pixels)**	0.79	9.92	<0.0001

Model 3 (*AIC* = −534.7; *R*^2^_adj_ = 0.51; *F*[4,95] = 26.75; *p* < 0.0001)^8^			

Shape (curved)	−0.11	−1.09	0.278
1st-order entropy	0.04	0.36	0.718
2nd-order entropy (20–240 pixels)	−0.11	−0.85	0.397
**2nd-order entropy (>240 pixels)**	0.76	9.24	<0.0001

Model 4 (*AIC* = −465.7; *R*^2^_adj_ = 0; *F*[l,98] = 0.48; *p* = 0.49)^9^			

Shape (curved)	−0.07	−0.70	0.487

*Model 1 describes the effect of shape (curved or angular) and the four image properties on the preferences ratings. Model 3 includes shape and the two measures of edge orientation entropy as variables. The two measures of entropy are the only predictors in Model 2. Shape is the only predictor in Model 4. Variables that are significant predictors when controlling for the remaining variables are highlighted by bold letters. ^1^Different from Model 1 (F[96,93] = 19.52, p < 0.0001) and Model 2 (F[96,95] = 11.55, p = 0.001). ^2^Different from Model 1 (F[95,93] = 21.06, p < 0.0001). ^3^Different from Model 1 (F[98,93] = 23.55, p < 0.0001), Model 2 (F[98,95] = 17.72, p < 0.0001), and Model 3 (F[98,96] = 18.75, p < 0.0001). ^4^Different from Model 1 (F[96,93] = 30.13, p < 0.0001) and Model 3 (F[96,95] = 19.08, p < 0.0001). ^5^Different from Model 1 (F[95,93] = 29.86, p < 0.0001). ^6^Different from Model 1 (F[98,93] = 13.63, p < 0.0001). ^7^Different from Model 1 (F[96,93] = 25.39, p < 0.0001). ^8^Different from Model 1 (F[95,93] = 37.03, p < 0.0001). ^9^Different from Model 1 (F[98,93] = 52.10, p < 0.0001), Model 2 (F[98,95] = 35.33, p < 0.0001), and Model 3 (F[98,96] = 52.31, p < 0.0001).*

For *interesting*, the full Model 1 explained 55% of the rating. The explained variance of *shape* alone (Model 4) did not differ significantly from Model 2 (entropies alone), but Model 2 explained less of the rating variability (14%) than the model that accounted for *shape* and the entropies (27%, Model 3).

For *harmonious*, self-similarity was the strongest predictor in the full Model 1. In Models 2 and 3, 2nd-order entropy (>240 pixels distance) was by far the strongest predictor while the effect of *shape* on the rating was weak. *Shape* alone (Model 4) did not predict the *harmonious* rating.

**Figure 8** confirms the linear dependence of the ratings for *pleasing* and *harmonious* on 2nd-order entropy (>240 pixel distance; *black* lines in **Figures 8G,I**). This dependence is also observed when angular and curved stimuli are analyzed separately (*red* lines and *blue* lines, respectively, in **Figures 8G,I**). For *interesting*, a correlation is obtained for curved stimuli only (*blue* line in **Figure 8H**). The values for the other entropies are close to their maximum value (about 4.585; vertical dashed lines in **Figures 8A–F**) and the observed correlations for *pleasing* and *harmonious* are negative and relatively weak (*black* lines in **Figures 8A,B,D,E**).

### Discussion

In Experiment 6, we generated a series of angular and curved line patterns, which systematically varied in their 2nd-order entropy. A closer inspection of these patterns revealed that the variability of 2nd-order entropy was driven mainly by edge pairs that were lying at the periphery of the patterns, i.e., for edge pairs that were spaced >240 pixels apart (**Figure 6**). Entropy was close to the maximum value for less distant edge pairs (20–240 pixels distance).

In view of the general preference for curvilinear patterns, we expected that the curved stimuli would be rated more highly than the angular patterns. We can dismiss this expectation for the rating of *harmonious* where *shape* alone (Model 4) was not a significant predictor. For *harmonious*, *shape* played only a minor role in the other regression models (Models 1 and 3; **Table 7**). Instead, 2nd-order entropy (>240 pixels distance) explained about half of the variability in the ratings (**Table 7** and **Figure 8I**). For the other two rating terms (*pleasing* and *interesting*), the results were less striking. Here, *shape* contributed 21% to explaining the variability in the rating of *pleasing* (Model 4) while the entropies (Model 2) explained 42% of the *pleasing* ratings. For *interesting*, the opposite pattern was found (26% of variance explained by Model 4, and 14% by Model 2). For *interesting*, edge density proved to be the strongest predictor in the full Model 1.

In conclusion, Experiment 6 indicates that the edge-orientation entropies are more powerful predictors for ratings of *pleasing* and *harmonious* than curvilinearity.

## General Discussion

We studied whether objective image properties predict aesthetic preferences in a wide variety of man-made visual stimuli, ranging from single closed contours, to photographs of every-day architectural patterns (building facades and interior scenes), and to music album covers, which are designed to attract the potential listeners’ attention. A particular focus of our study was on the question of whether edge-orientation entropy and curvilinearity have overlapping effects on aesthetic ratings.

Our results demonstrate that edge-orientation entropy affects ratings to different degrees, depending on the image category. The largest effect was observed for the facade photographs (Experiment 3) and for the complex artificial line patterns (Experiment 6), where up to half of the variance in the aesthetic ratings is accounted for by 1st-order or 2nd-order edge-orientation entropy. By contrast, the two entropy variables accounted for only 3.5% of the variance in the rating of the music covers for *harmonious*. In the following sections, we will first discuss these differences between the stimuli in more detail. Second, we will compare the effect of the different variables, especially with respect to the difference between edge-orientation entropy and curvilinearity, on the aesthetic ratings. Third, the difference between 1st-order entropy and 2nd-order entropy is evaluated. Fourth, we will point out differences between the rating terms in their dependence on the image properties. Finally, we will discuss some shortcomings of our study and possible future directions of research.

### Differences Between the Visual Stimuli and How Their Rating Is Predicted by Image Properties

All image properties calculated in the present study represent global visual features that relate to the formal structure of the images. Our results confirm previous studies that revealed an effect of such global image properties on the preference of visual stimuli (Berlyne, 1974; Taylor, 2002; Jacobsen and Höfel, 2002; Spehar et al., 2003; Bar and Neta, 2006; Graham and Redies, 2010; Forsythe et al., 2011; Bertamini et al., 2016; Gómez-Puerto et al., 2016; Brachmann and Redies, 2017). However, it is generally agreed that not only image structure, but also the displayed content can determine the aesthetic preference of visual stimuli (Bullot and Reber, 2013; Redies, 2015; Pelowski et al., 2017). We therefore expected that formal image properties would affect aesthetic ratings less strongly for images that display recognizable content (Experiments 3–5) than for images that are devoid of semantic meaning (i.e., the abstract patterns in Experiments 1, 2, and 6). Our results for the different image categories largely confirm this expectation. On the one hand, for the abstract patterns, all image properties together (Model 1) explained up to 75% of the rating variability (**Tables 2**, **3**, **7**). The lowest explained variability (13%) was obtained for the *pleasing* rating of the Taprats patterns (**Table 3**). On the other hand, explained variance for the *pleasing* and *harmonious* ratings of the music covers were below 10% (lowest value, 3.5%; **Table 6**). Although we did not measure the effect of displayed content by objective means in Experiment 5 (music album covers), it is possible that the observers’ preferences for particular music genres or individual musicians might elicit a content-based bias, thereby diminishing the effect of formal image properties. A spillover effect for preference in the other direction, i.e., from the visual to the auditory domain, has been demonstrated for music-related material previously (Libeks and Turnbull, 2011). For the *interesting* rating of music covers, the predicted variance was higher (25%), but it was driven mainly by *metal* (**Table 6**), possibly because many covers of metal music show exciting, shocking or even disgusting content, which arouses the observers’ interest. For the interior scenes, predicted variance was also low (around 15% for the *pleasantness* and *beauty* ratings; **Table 5**). By contrast, the image properties predict the rating of the building facades to a higher degree (29–55% of the variance predicted). Similar to the abstract stimuli, the facade images are likely to be rated based on their visual structure rather than on contextual factors, such as familiarity with or explicit knowledge about the style or the designer of the decorations. We thus conclude that the image properties studied, including the edge-orientation entropies, can be strong predictors for preference, especially for abstract stimuli or stimuli that are evaluated predominantly based on their visual structure.

### Differences Between the Image Properties and How They Affect the Ratings

In order to study differences in the effects of the image properties on the aesthetic ratings, we compared a full model (Model 1), which comprised all measured properties as independent variables, with models restricted to a subset of these properties. As outlined in the Introduction, we were particularly interested in the effect of the two measures of entropy, also in comparison to curvilinearity (*shape* or *contour* variables), in Experiments 1, 4, and 6.

First, we compared the full model with all variables (Model 1) to Model 3, which omitted self-similarity and edge density and was thus restricted to the two edge-orientation entropies (Experiments 2, 3, and 5) plus the curvilinearity variables (*shape/contour*; Experiments 1, 4, and 6; **Table 1**). The predicted variance (*R*^2^_adj_) shows a decrease and/or the *AIC* value an increase from Model 1 to Model 3, which indicates that the omitted factors (edge density and self-similarity) have an additional independent effect on the ratings (except for Experiment 1 and *pleasing* in Experiment 5). The relative decrease from Model 1 to Model 3 was small for facade photographs (e.g., from 55 to 48% for the *pleasing* rating) and the complex line patterns in Experiment 6 (e.g., from 63 to 48% for the *pleasing* rating), but larger for the interior scenes (e.g., from 14 to 4.4% for the *pleasantness* rating) and strongest for the *harmonious* rating of the Taprats images (from 43 to 13%). Importantly, except for the *interesting* rating of the album covers, the edge-orientation entropies and the curvilinearity variables (*shape/contour)* together (Model 3) remained significant predictors for all aesthetic ratings in all image categories. This finding underscores the importance of these measures for aesthetic evaluations of different types of man-made visual stimuli.

Second, we compared the predictive power of the edge-orientation entropies with that of the curvilinearity variables (*shape/contour*) in Experiments 1, 4, and 6 (**Table 1**). For the interior scene photographs (Experiment 4), *contour* alone did not contribute to the *pleasantness* and *beauty* ratings (unlike the architectural variable *openness*, **Table 5**). Moreover, *shape* alone did not contribute to the *harmonious* rating of the complex line patterns in Experiment 6 (**Table 7**). The predicted variance in the *pleasing* rating increased from 42 to 48% and the variance in the *interesting* rating increased from 14 to 27%, when *shape* was added (Model 3 in **Table 7**) to the model that consists of the edge-orientation entropies only (Model 2). Similarly, for the rating in Experiment 1, *shape* contributed an additional 17% to the variance predicted by the entropies alone (58%; compare Models 2 and 3 in **Table 2**). When Model 2 (entropies alone) was compared to Model 4 (*shape* alone), the entropies alone predicted more of the rating variance for the *pleasing* and the *harmonious* ratings in Experiment 6 than *shape* alone. Strikingly, there was no significant difference between the two models for the other ratings in Experiments 1 and 6 (**Tables 2**, **7**).

Together, these results suggest that edge-orientation entropies are as good or better predictors for the aesthetic ratings than the curvilinearity variables (*curved/angular*). This is particularly evident in Experiment 6 where the angular line patterns are rated as more *pleasing* and *interesting* than the curved ones on average. Here, 40 angular or curved lines are superimposed in a texture-like arrangement. It is possible that edge-orientation entropy is a strong predictor for the preference of such textures, while curved or angular shape is more important for the preference of lines that are perceived individually. More experiments are needed to address this question.

Moreover, the edge-orientation entropies share a large portion of predicted variance for the preference for curved over angular stimuli. In other words, for most of the ratings in Experiments 1, 4, and 6, the entropies can substitute for the *curvilinearity* variable *(shape or contour)*, at least in part, to predict the aesthetic ratings. Thus, the question of whether stimuli are curved or angular can be partly operationalized by measuring edge-orientation entropy in the images. In view of the uncertainties that underlie the terminology and concepts of the *curved/angular* account (Gómez-Puerto et al., 2016), the concept of edge-orientation entropy may thus have the advantage that it is captured more precisely in mathematical terms (Geisler, 2008; Redies et al., 2017). However, whether a visual stimulus is curved or angular, can be more easily grasped by intuition.

### First-Order and 2nd-Order Edge-Orientation Entropy

As explained in more detail in the Section “General Methods,” 1st-order edge-orientation entropy is a measure of how uniformly the edge orientations are distributed across the full spectrum of orientations in an image. Second-order edge-orientation entropy is a measure of how independent edge orientations are distributed across an image. Both measures have an upper bound that depends on the number of orientation bins analyzed (≈ 4.585 for the 24 bins analyzed in the present study). In a previous study, we showed that large subsets of traditional artworks assume values close to this upper bound (Redies et al., 2017). In the present study, the edge orientation entropies of some image categories (e.g., of music cover designs) are also close to the upper bound, whereas those of other image categories (e.g., photographs of facades) scatter more widely. In general, we observe strong and positive predictive effects of the entropies on the aesthetic ratings if the entropy values scatter more widely. This pattern is observed for the simple shapes (**Figures 1E,F**), the facade images (**Figures 3F–K**), the interior scenes (**Figures 4E–H**), and for the 2nd-order entropy (>240 pixels) in Experiment 6 (**Figures 8G–I**). Vice versa, we observe weak and, in some cases, even negative predictive effects of the entropies for image categories with entropy values that are clustered close to the upper bound, as is the case for the album covers (**Figures 5D–I**), and for short-range 1st-order entropy and 2nd-order entropy (20–240 pixels) in Experiment 6 (**Figures 8A–F**). The only exception is the *harmonious* rating (but not the *interesting* and *pleasing* ratings) of the *Taprats* images (**Figure 2J**), where 2nd-order entropy is a negative predictor despite its wide range of values. In this case, however, self-similarity is a relatively strong predictor for the *harmonious* rating (**Figure 2K** and **Table 3**), compared to 2nd-order entropy. We thus conclude that, with this exception, the two measures of entropy are positively associated with aesthetic ratings, and that this effect is stronger if the range of entropy values is wide and remote from its upper bound.

The relative contribution of 1st-order entropy and 2nd-order entropy to the aesthetic ratings differs between the image categories. In some cases, e.g., for the building facades, 1st-order entropy is a stronger predictor for the aesthetic ratings than 2nd-order entropy (**Table 4**). In other cases, e.g., for the complex line patterns in Experiment 6, 2nd-order entropy has a stronger effect on the aesthetic rating that 1st-order entropy (**Table 7**). It should be pointed out that the two measures of entropy are not independent of each other (Redies et al., 2017). The Spearman correlations for 1st-order entropy and 2nd-order entropy (>240 pixels) range from a correlation coefficient of *r* = 0.97 (*p* < 0.0001) for the building facades (Experiment 3) to no correlation at all in Experiment 6. It is thus difficult to extract a common overall pattern for the differential effect of the entropies on the ratings.

### Differences Between Rating Terms and Their Dependence on the Image Properties

As outlined in the Introduction, the three ratings terms used in Experiments 2, 3, 5, and 6 mirror different aesthetic aspects of the stimuli. *Pleasing* has been described as a more subjective judgment that combines emotional arousal and aesthetic effectance while *interesting* is thought to reflect a more objective way to evaluate images (Cupchik and Gebotys, 1990); *harmonious* was proposed to relate to the hedonic value of the image composition (Redies et al., 2015). There may be differences, however, in what exactly is considered *pleasing*, *interesting*, or *harmonious* in various types of images, for example, between abstract line images and music album covers. It is therefore not surprising to see differential effects of the image properties on the ratings in Experiments 2, 3, 5, and 6. When we evaluated the full model (Model 1), we did not observe any systematic variation of the effect of the image properties on the ratings across all image categories. For example, Model 1 predicted a larger percentage of the *harmonious* rating for the Taprats images than for the *pleasing* and *interesting* ratings (**Table 3**), but the inverse pattern was seen for the facade images (**Table 4**), while the percentage predicted was rather similar for all ratings on the complex line stimuli (**Table 7**). Interestingly, for the facade images, self-similarity was positively related to *harmonious* but negatively to *pleasing* and *interesting*. We have observed a similar difference for complex abstract images, where images with higher self-similarity were rated more *harmonious* but less *interesting* (Redies et al., 2015). In conclusion, the proportion of rating variance that is predicted by the image properties depends not only on the image properties but also on the rating terms.

## General Conclusions and Experimental Limitations

Previously, we have shown that edge-orientation entropy is high in traditional artworks of different cultural provenance when compared to many categories of other man-made or natural patterns and scenes (Redies et al., 2017). The present results indicate that edge-orientation entropy predicts aesthetic ratings in diverse artificial images and photographs of man-made scenes. The magnitude of this effect, however, depends on the category of images analyzed, on the range of entropy values encountered, and on the type of aesthetic ratings (*pleasing, interesting*, or *harmonious*). In general, higher edge-orientation entropies correlate with higher aesthetic rating, in particular, if entropy values cover a wide range and do not approach their upper bound. Moreover, it should be noted that the findings of the present study are specific to the laboratory setting (Brieber et al., 2015) and our analysis was restricted to six special types of man-made images. Because aesthetic ratings can be domain-specific (Hayn-Leichsenring et al., 2013; Jacobsen, 2014), it remains to be studied whether our findings can be generalized to other man-made images or to natural patterns or scenes. Also, the participants in our study were undergraduates or had finished higher education; all participants were younger than 40 years. These limitations and any idiosyncratic taste associated with them may have had an effect on the ratings, especially for the images that displays cultural content (interior scenes and music album covers).

Despite these caveats, the present findings lead us to speculate that images with high edge-orientation entropy are aesthetically preferred over ones with low entropy. Results from Experiment 6, where we systematically manipulated 2nd-order entropy in artificial stimuli that were composed of otherwise identical line elements, suggests that this effect is a causal one. Here, the edge orientation entropies alone are strong predictors of the *pleasing* and *harmonious* ratings (**Table 7** and **Figures 8G,I**). Moreover, edge orientation entropy is a predictor for the ratings that is as strong or stronger than their curved or angular *shape/contour* (compare Models 2 and 4 in **Tables 2**, **7**), with a large degree of overlap of predictive power between these variables (compare Models 2 and 3 in **Tables 2**, **7**).

It would be interesting to study whether the visual preference for high edge-orientation entropy is shared in different cultures or even in species like great apes, as has been shown for curvilinear patterns (Munar et al., 2015; Gómez-Puerto et al., 2017). Despite the widespread preference for curvature, Cotter et al. (2017) described inter-individual differences that can modulate this preference. In particular, participants with higher artistic expertise or openness to experience showed a stronger preference for smooth curvatures in irregular polygons. Several other studies have previously revealed differences between human observers in evaluating the hedonic values of visual stimuli (Berlyne, 1971; Jacobsen and Höfel, 2002; Palmer and Griscom, 2012). For example, individual differences in rating studies on visual complexity have been reported (e.g., Spehar et al., 2015, 2016; Bies et al., 2016; Güclütürk et al., 2016). Mallon et al. (2014) described that clusters of participants preferred different combinations of low-level image properties, such as color characteristics and self-similarity. To obtain an overview of the influence of edge orientation entropy on preference for different sets of stimuli, the present study focuses on group-level differences. It thus remains to be studied whether the preference for high edge orientation entropy is subject to inter-individual variability.

## Author Contributions

MG and CR conceived the study and contributed stimuli. AB wrote the computer code to generate the stimuli for Experiment 6. MB contributed the stimuli for Experiment 1. MG and AK carried out the experiments. MG and CR analyzed the data. MG, AB, and MB contributed to writing the paper. CR wrote the paper and supervised the study at all stages.

## Conflict of Interest Statement

The authors declare that the research was conducted in the absence of any commercial or financial relationships that could be construed as a potential conflict of interest.

## Footnotes

1 http://www.cgl.uwaterloo.ca/csk/washington/taprats (last accessed 28 December 2017)
