# Supplementary material for: Edge-Orientation Entropy Predicts Preference for Diverse Types of Man-Made Images
Source: Front Neurosci. 2018 Sep 28;12:678. doi: 10.3389/fnins.2018.00678 (PMC6172329; doi:10.3389/fnins.2018.00678)
Supplement: Supplementary file 1 [file Table_1.pdf]

Supplementary Material

Edge-Orientation Entropy Predicts Preference for Diverse Types of Man-Made Images

Maria Grebenkina, Anselm Brachmann, Marco Bertamini, Ali Kaduhm, Christoph Redies*

*** Correspondence:** Corresponding Author: christoph.redies@med.uni-jena.de

Supplementary Table 1. Results for individual images and median results for the *Taprats* stimuli used in Experiment 2. The values listed are for the images shown in Figure 2A-F, as well as the *median* value (± *median absolute deviation* [*MAD*]) for all 100 images.

	Statistical image properties				Ratings		
Images	1st-order entropy	2nd-order entropy	Edge density	Self-similarity	*Pleasing*	*Interesting*	*Harmonious*
A	4.45	4.44	119.6	0.65	0.65	0.67	0.42
B	3.61	4.16	238.0	0.62	0.76	0.74	0.57
C	4.45	4.12	146.8	0.87	0.45	0.37	0.72
D	4.07	4.16	75.0	0.98	0.31	0.32	0.50
E	4.29	3.91	71.7	0.84	0.34	0.25	0.63
F	4.52	4.36	135.7	0.60	0.41	0.43	0.30
Median	4.50	4.34	196.9	0.88	0.46	0.46	0.58
± *MAD*	± 0.06	± 0.17	± 88.8	± 0.07	± 0.06	± 0.07	± 0.08

Supplementary Table 2. Results for individual images and median results for the facade photographs (Experiment 3). The values listed are for the images shown in Figure 3A-E, as well as for all 50 images (*median* ± *median absolute deviation* [*MAD*]).

Images	Statistical image properties				Ratings		
	1st-order entropy	2nd-order entropy	Edge density	Self-similarity	*Pleasing*	*Interesting*	*Harmonious*
A	3.49	4.15	121.5	0.76	0.71	0.73	0.77
B	3.62	4.11	171.6	0.75	0.71	0.65	0.75
C	2.97	3.65	167.2	0.79	0.46	0.39	0.52
D	2.20	2.67	140.4	0.78	0.23	0.24	0.41
E	2.38	3.04	99.2	0.77	0.19	0.22	0.31
Median	3.04	3.82	140.7	0.76	0.52	0.49	0.52
± *MAD*	± 0.39	± 0.27	± 21.1	± 0.03	± 0.12	± 0.14	± 0.09

Supplementary Table 3. Results for individual images and median results for the interior scene photographs (Experiment 4). The values listed are for the images shown in Figure 4A-D, as well as for all 200 images (*median* ± median absolute deviation [*MAD*]). Abbreviations: 1st, 1st-order entropy; 2nd, 2nd-order entropy; cl, closed space; hc, high ceiling; lc, low ceiling; op, open space; ro, round contour; sq, square contour.

	Statistical image properties				Ratings	
Images	1st-order entropy	2nd-order entropy	Edge density	Self-similarity	Pleasantness	Beauty
A (hc, op, ro)	3.79	4.32	133.5	0.65	4.50	4.28
B (lc, op, sq)	3.81	4.25	70.6	0.55	4.22	4.22
C (lc, cl, sq)	4.08	4.34	61.4	0.55	2.89	3.17
D (lc, cl, sq)	3.03	3.67	57.2	0.56	1.17	1.06
Median	3.85	4.29	76.3	0.56	3.00	2.89
± *MAD*	± 0.25	± 0.10	± 19.8	± 0.06	± 0.61	± 0.61

Supplementary Table 4. Results for the music album covers (Experiment 5). The values listed are for the exemplary images shown in Figure 5A-C, as well as for 50 images each of the three album categories (*median* ± *median absolute deviation [MAD]*).

	Statistical image properties				Ratings		
Images	1st-order entropy	2nd-order entropy	Edge density	Self-similarity	*Pleasing*	*Interesting*	*Harmonious*
A (pop)	4.48	4.51	81.1	0.67			
B (metal)	4.49	4.52	98.0	0.69			
C (classic)	4.22	4.48	58.0	0.55			
Pop	4.35 ± 0.11	4.48 ± 0.03	99.9 ± 21.3	0.60 ± 0.07	0.42 ± 0.10	0.36 ± 0.14	0.45[5] ± 0.08
Metal	4.41[1] ± 0.10	4.51 ± 0.03	103.7 ± 27.1	0.66[2] ± 0.08	0.40[3] ± 0.06	0.50[4] ± 0.08	0.43 ± 0.07
Classic	4.30 ± 0.11	4.49 ± 0.04	102.2 ± 23.5	0.63 ± 0.06	0.38 ± 0.07	0.31 ± 0.10	0.49 ± 0.08

[1] different from *classic* (p<.01; Kruskal-Wallis test with Dunn's post-test)

[2] different from *pop* (p<.01) and *classic* (p<.05)

[3] different from *classic* (p<.05)

[4] different from *pop* (p<.001) and *classic* (p<.0001)

[5] different from *metal* and *classic* (both p<.0001)

Supplementary Table 5. Results for the stimuli used in Experiment 6. The values listed are for the exemplary images shown in Figure 6A-C, as well as for 50 images each of the curved and angular categories (*median ± median absolute deviation [MAD]*).

Images	Statistical image properties					Ratings		
	1st-order entropy	2nd-order entropy (20-240 pixels)	2nd-order entropy (>240 pixels)	Edge density	Self-similarity	*Pleasing*	*Interesting*	*Harmonious*
A (angular)	4.28	4.34	3.21	150.0	0.50	0.37	0.51	0.34
B (angular)	4.48	4.46	3.79	166.6	0.57	0.37	0.41	0.38
C (angular)	4.43	4.43	4.09	179.7	0.60	0.53	0.47	0.62
D (curved)	4.54	4.49	3.48	123.9	0.54	0.23	0.31	0.31
E (curved)	4.51	4.49	3.90	165.2	0.60	0.35	0.44	0.35
F (curved)	4.53	4.48	4.15	175.9	0.62	0.45	0.45	0.50
Angular	4.49[1]	4.44[1]	3.77	165.0	0.54	0.38[1]	0.46[1]	0.38
	± 0.034	± 0.011	± 0.22	± 6.43	± 0.037	± 0.028	± 0.026	± 0.67
Curved	4.54	4.48	3.80	165.8	0.54	0.34	0.41	0.35
	± 0.017	± 0.007	± 0.19	± 5.63	± 0.026	± 0.037	± 0.031	± 0.064

[1] significantly different from *curved* (p<.0001; two-tailed Mann-Whitney test)
